# Multi-Omics Approach in the Identification of Potential Therapeutic Biomolecule for COVID-19

**DOI:** 10.3389/fphar.2021.652335

**Published:** 2021-05-12

**Authors:** Rachana Singh, Pradhyumna Kumar Singh, Rajnish Kumar, Md. Tanvir Kabir, Mohammad Amjad Kamal, Abdur Rauf, Ghadeer M. Albadrani, Amany A. Sayed, Shaker A. Mousa, Mohamed M. Abdel-Daim, Md. Sahab Uddin

**Affiliations:** ^1^ Amity Institute of Biotechnology, Amity University Uttar Pradesh, Lucknow Campus, Lucknow, India; ^2^ Plant Molecular Biology and Biotechnology Division, Council of Scientific and Industrial Research- National Botanical Research Institute (CSIR-NBRI), Lucknow, India; ^3^ Department of Pharmacy, Brac University, Dhaka, Bangladesh; ^4^ West China School of Nursing/Institutes for Systems Genetics, Frontiers Science Center for Disease-related Molecular Network, West China Hospital, Sichuan University, Chengdu, China; ^5^ King Fahd Medical Research Center, King Abdulaziz University, Jeddah, Saudi Arabia; ^6^ Enzymoics, Novel Global Community Educational Foundation, Hebersham, NSW, Australia; ^7^ Department of Chemistry, University of Swabi, Khyber Pakhtunkhwa, Pakistan; ^8^ Department of Biology, College of Science, Princess Nourah bint Abdulrahman University, Riyadh, Saudi Arabia; ^9^ Zoology Department, Faculty of Science, Cairo University, Giza, Egypt; ^10^ Pharmaceutical Research Institute, Albany College of Pharmacy and Health Sciences, Rensselaer, NY, United States; ^11^ Pharmacology Department, Faculty of Veterinary Medicine, Suez Canal University, Ismailia, Egypt; ^12^ Department of Pharmacy, Southeast University, Dhaka, Bangladesh; ^13^ Pharmakon Neuroscience Research Network, Dhaka, Bangladesh

**Keywords:** COVID-19, SARS-CoV-2, coronavirus, multi-omics, biomolecules, therapeutic molecules

## Abstract

COVID-19 is caused by severe acute respiratory syndrome coronavirus 2 (SARS-CoV-2). It has a disastrous effect on mankind due to the contagious and rapid nature of its spread. Although vaccines for SARS-CoV-2 have been successfully developed, the proven, effective, and specific therapeutic molecules are yet to be identified for the treatment. The repurposing of existing drugs and recognition of new medicines are continuously in progress. Efforts are being made to single out plant-based novel therapeutic compounds. As a result, some of these biomolecules are in their testing phase. During these efforts, the whole-genome sequencing of SARS-CoV-2 has given the direction to explore the omics systems and approaches to overcome this unprecedented health challenge globally. Genome, proteome, and metagenome sequence analyses have helped identify virus nature, thereby assisting in understanding the molecular mechanism, structural understanding, and disease propagation. The multi-omics approaches offer various tools and strategies for identifying potential therapeutic biomolecules for COVID-19 and exploring the plants producing biomolecules that can be used as biopharmaceutical products. This review explores the available multi-omics approaches and their scope to investigate the therapeutic promises of plant-based biomolecules in treating SARS-CoV-2 infection.

## Introduction

The world has faced several chronic diseases, a few epidemics, and pandemics that were disastrous and eliminated a predominant human population throughout history. Among all these, the present-day COVID-19 is potentially contagious and has left the most devastating effect on humankind (Prasad and Prasad, 2020; Shahriar et al., 2021). The virulent nature of COVID-19 and the rise of its different strains have forced researchers worldwide to search for rapid diagnostic methods for detection, development of new vaccines, various therapeutic drugs, and immunization options. Success has been achieved in developing different diagnosis methods, but no drug has been developed to efficiently provide treatment to the infected patient (Hossain et al., 2020; Lurie et al., 2020; Loeffelholz and Tang, 2020).

Coronavirus belongs to the family Coronaviridae in the order Nidovirales. This group of viruses is classified into four genera such as *Alpha coronavirus* (α-CoV), *Beta coronavirus* (β-CoV), *Gamma coronavirus* (γ-CoV), and *Delta coronavirus* (δ-CoV) (Woo et al., 2010). Both the alpha- and beta-coronaviruses tend to infect mammals. Among them, *β*-CoV has become the utmost concern of the world due to its ability to cause serious illness in the human population, like the Middle East respiratory syndrome–related coronavirus (MERS-CoV), SARS-CoV, and SARS-CoV-2, which cause fatal respiratory tract infection in humans (Lu et al., 2020). The structural and nonstructural information of SARS-CoV-2 has already been explained and explored by many studies available in the literature (Hillen et al., 2020; Jin et al., 2020; Clark et al., 2021; Hasana et al., 2021; Semper et al., 2021).

Although many vaccines such as Comirnaty (mRNA based), Moderna (mRNA based), AstraZeneca/Covishield (adenovirus vaccine), Sputnik V (nonreplicating viral vector), CoronaVac (inactivated vaccine), BBIBP-CorV (inactivated vaccine), EpiVacCorona (peptide vaccine), Convidicea (adenovirus type 5 vector-recombinant vaccine), and Covaxin (inactivated vaccine) have been approved in various countries, a specific approved drug for COVID-19 is still missing. The significant challenges in mass immunization include large-scale production, cost-effectiveness, and global allocation. Moreover, until the vaccination preferably drives herd immunity, social distancing is still the most effective measure adopted all over the world to prevent the spread of the disease (McKibbin and Fernando, 2020; Sohrabi et al., 2020). Mostly, preexisting antiviral drugs are still being used in the management of SARS-CoV-2–infected patients. Simultaneously, the repurposing of drugs is also recommended for testing and application. Some preexisting antiviral drugs like remdesivir and lopinavir/ritonavir are currently being used in the management of COVID-19 (Kumar et al., 2020a; Choy et al., 2020; Wang et al., 2020f; Zhu et al., 2020).

However, there is an urgent need to investigate and discover potential therapeutic compounds that could restrict viral replication and its assembly in the human body. Finding a permissive cell and delivering genetic information into its cytoplasm are essential steps for viral infection into the host cell. From a therapeutic point of view, there are at least two important ways. The first is that blocking viral entry stops infection early on and the second is preventing viral replication. The antiviral drugs can be developed by targeting different viral entry stages, either by blocking virus-specific interactions or by inhibiting conserved cellular mechanisms that viruses exploit to enter cells (Mazzon and Marsh, 2019).

Advancements in biotechnology and computational analysis have taken place in the past decade, and they have provided massive amounts of new data that have given an optimistic outlook for intensifying disease treatment developments (Belfiore et al., 2020; Infusino et al., 2020; Stebbing et al., 2020). Nearly every disease and clinical research area has exploded with enormous data (Lin et al., 2020). Computational analysis of the available diverse omics data could provide an in-depth understanding of molecular mechanisms and associated transitions of the diseases (Kumar et al., 2020b; Muthuramalingam et al., 2020; Su et al., 2020). Clinical data management, genome and proteome analyses, next-generation sequencing data mining, machine learning, and deep learning algorithms have progressed significantly for mining patterns from such enormous data (Kumar et al., 2016; Kumar et al., 2018; Hossain et al., 2021; Overmyer et al., 2021).

**FIGURE 1:**
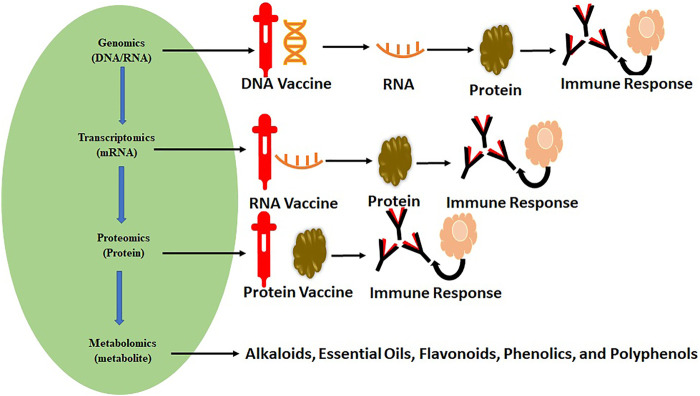
Omics science and flow of biological information assisting the vaccine development and drug repurposing for various diseases.

**FIGURE 2:**
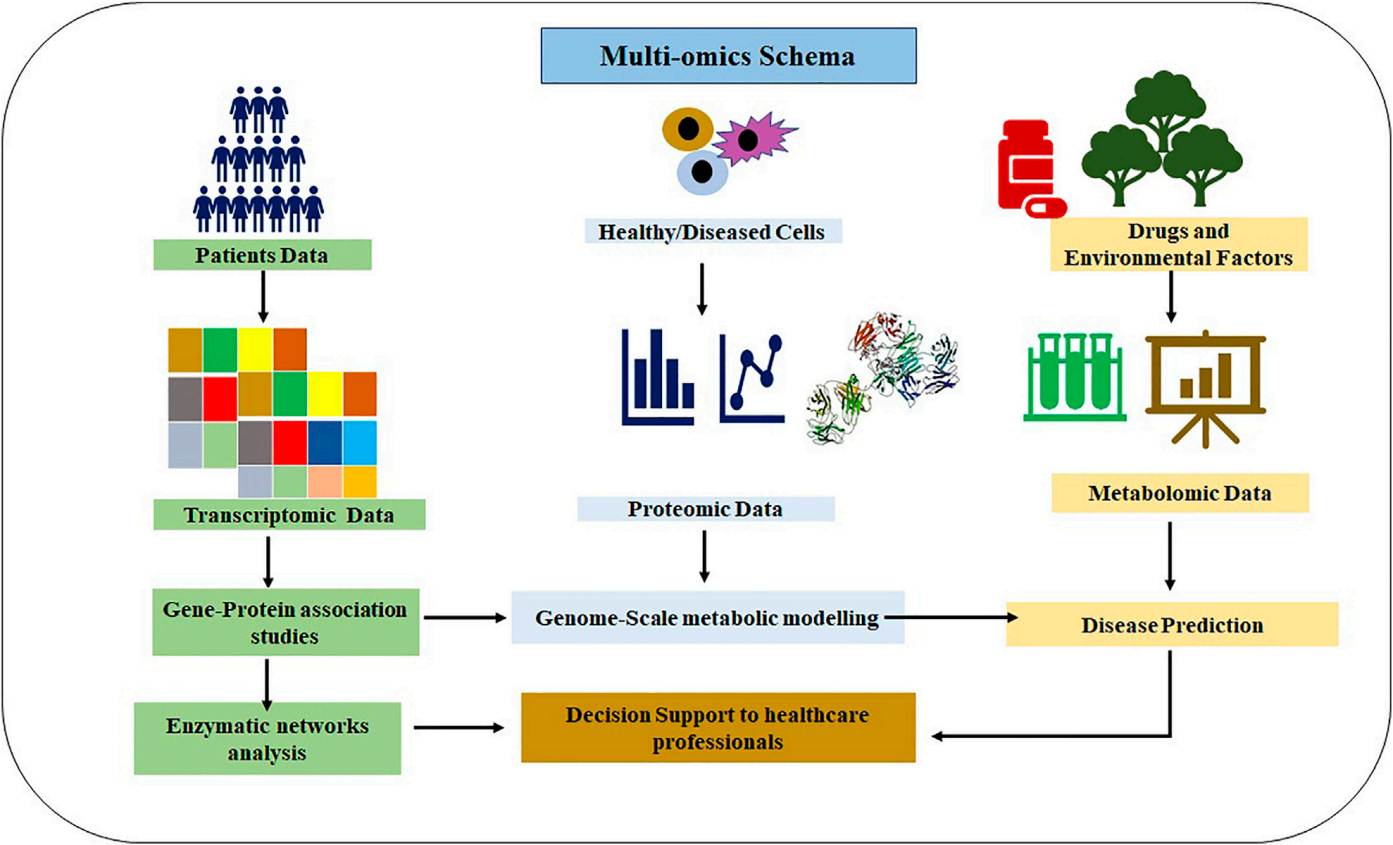
Multi-omics schema integrating different types of omics data to support the healthcare professionals in decision-making.

Omics represent the collective technologies that help to investigate the different molecules’ roles and actions that make up an organism’s cells. These include genomics (gene), transcriptomics (mRNA), proteomics (proteins), and metabolomics (metabolites). These techniques have already played a significant role in vaccine development and repurposing of drugs, as shown in Figure 1. Omics technologies’ main importance lies in detecting and verifying all gene products (transcripts, proteins, and metabolites) available in any given biological sample. Omics analysis has been extensively applied in drug discovery (Horgan and Kenny, 2011; Chen B. et al., 2020; Aaron et al., 2020) and estimation of their effectiveness and toxicity (Figure 2). These are high-throughput technologies that have been significantly assisting in describing gene/protein expression profiles and their complex effects for SARS-CoV-2 (Wang et al., 2020a; Bojkova et al., 2020; Kim et al., 2020; Li et al., 2020). With their immense possibilities, the powerful omics techniques seem like they will continue supporting researchers and healthcare professionals in exploring and exploiting SARS-CoV-2 pathophysiology for a deeper understanding of its processes and understanding the disease for diagnosis, screening, and prognosis (Wang et al., 2020b; Hart and Halden, 2020; Robson, 2020).

The reports on the uses of preexisting drugs suggest that 3CLpro, Spike, RNA-dependent RNA polymerase (RdRp), PLpro, and human angiotensin-converting enzyme 2 (human ACE2) are potential drug targets for SARS-CoV-2 for further *in vitro* and *in vivo* studies (Xu et al., 2020).

**FIGURE 3:**
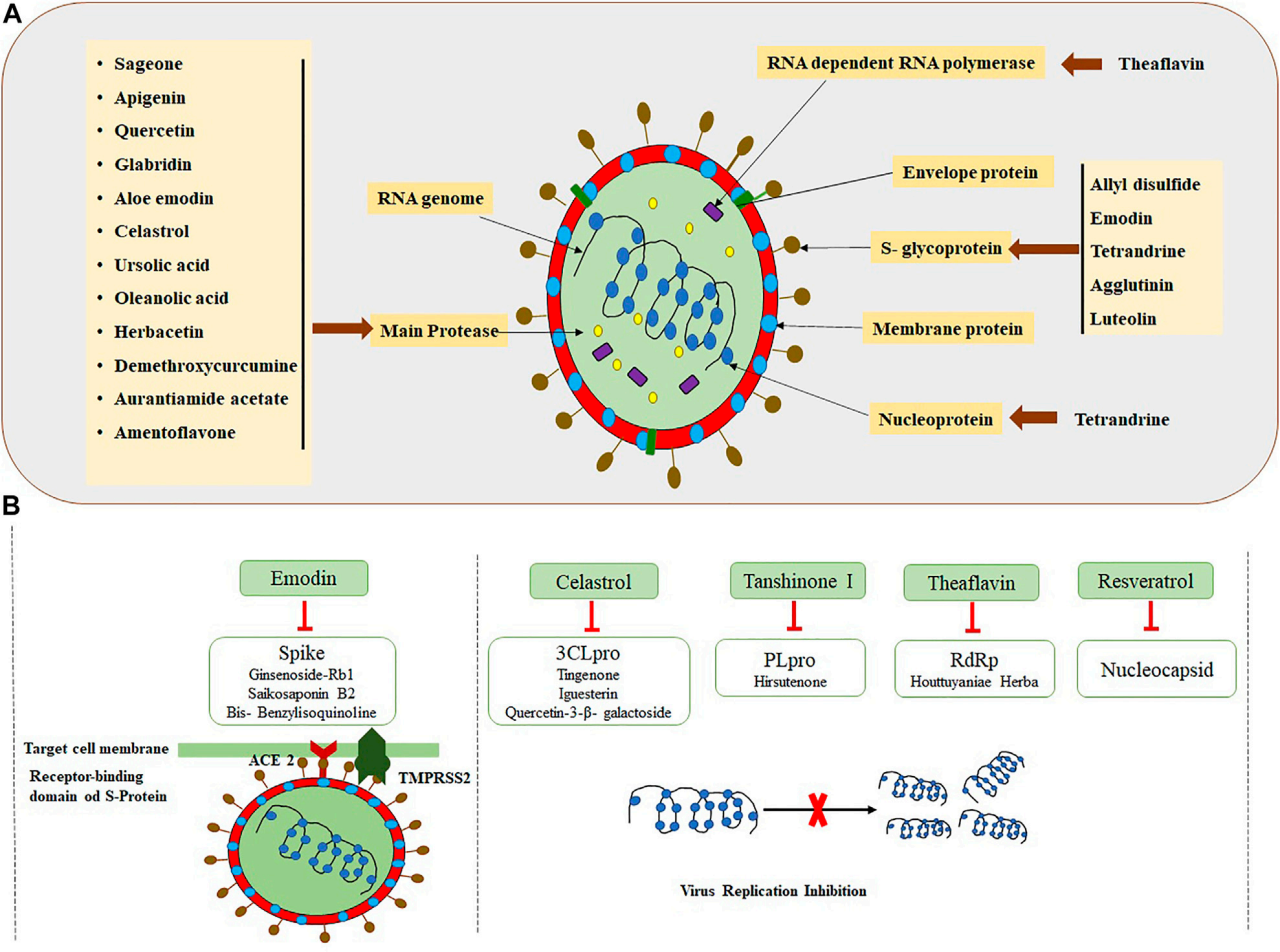
Interaction of SARS-CoV-2 structural proteins with the potential plant-based biomolecules. **(A)** Structure of SARS-CoV-2 and available biomolecules. **(B)** Rich resources of Chinese herbal medicines targeting SARS-CoV-2 (Sharma N. et al., 2020; Xian et al., 2020).

Since ancient times, plant preparations have been utilized as herbal medicines, which probably also contain active antiviral extracts/compounds, to cure and control infectious diseases (Israt and Ahmet, 2020; Yang et al., 2020; Al-Ishaq et al., 2020). Screening of plants to find the potential antiviral compounds was conducted long ago, during 1952. A total of 288 extracts from plant sources were experimented to check their role against the influenza A virus (Chantrill et al., 1952). Thereafter, many scientists have worked on utilizing the potential of plant extracts with different viral diseases. Debiaggi et al. (1988) worked on the antiviral activity of *Chamaecyparis lawsoniana* (A. Murr bis) Parl. against herpes simplex virus type 2. Another study found that *Geranium sanguineum* L. has an antiviral activity for the influenza A virus. It was discovered that polyphenolic extract derived from *Geranium sanguineum* L. effectively inhibits the influenza virus’s reproduction (Serkedjieva, 2003). Medicinal plants contain a variety of secondary metabolites with the potential to inhibit viral proteins and their enzymes, which consequently stop/suppress the viral entry and replication into the host (Semple et al., 1998; Li and Peng, 2013; Arbab et al., 2017; Akram et al., 2018; Dhama et al., 2018). Kotwal and coworkers studied the acidic extract of *Trifolium* species that showed an antiviral activity (broad-spectrum) (Kotwal et al., 2005). Several studies have been carried out, and they have confirmed that bioactive natural compounds are potential candidates for the treatment of SARS-CoV-2 because of their effective antiviral activity (Figure 3) (Behl et al., 2020; Kabir et al., 2020; Qamar et al., 2020; Zhang L. et al., 2020; Kumar et al., 2021).

These natural biomolecules are economical and deemed safe with minimum side effects compared to synthetic compounds (Enmozhi et al., 2020). Although the use of natural biomolecules generally involves minimal or no side effects, sometimes, they can potentially be toxic. This may be due to misidentification of the biomolecules in the form that they are sold, wrong preparation, dispensing, or administration by inadequately trained personnel (Karimi et al., 2015). Moreover, if a potential biomolecule could be found to regulate COVID-19 infection, it would be a boon in the treatment and could be used immediately after acquiring compulsory ethical clearances. Despite sufficient evidence that plant-derived biomolecules are effective as an antiviral agent, their use is still limited because these compounds are either available at minimal levels or might not be stable outside the plants (Anand et al., 2021; Balkrishna et al., 2021). Therefore, a comprehensive strategy is needed to properly identify potential biomolecules’ biosynthetic pathways and analyze the genes underlying those pathways (Shree et al., 2020). As the identification of plant-based moieties with anti–SARS-CoV-2 action is in a devolving phase, it will take a long time to explore their full potential using the traditional drug development method. The omics techniques used in plant molecular biology could accelerate this procedure using a strategically planned study design and provide a platform to researchers and the bio-manufacturer of these biomolecules to manage the COVID-19 pandemic (Sharma N. et al., 2020; Chojnacka et al., 2020). This article has explored the plant-based compounds that contain antiviral activities to assess the impact of the multi-omics approach for plant biomolecular research and its potential application against SARS-CoV-2.

## Potential of Plant-Derived Biomolecules for COVID-19

There is enough evidence that herbal medicines have been used in different parts of the world since prehistoric times. Asian countries like India, Japan, China, and Pakistan, and countries in Africa are using medicinal plants for herbal treatment. Herbal plants are supplemented with various phytochemicals like alkaloids, flavonoids, phenolic acids, lignins, and terpenoids. All have shown their potential against infectious diseases (Kim et al., 2019; Lalani and Poh, 2020). It was demonstrated that biomolecules of plants exhibit inhibitory activity on hepatitis-B virus (Huang et al., 2006), herpes simplex virus type-2 (Debiaggi et al., 1988), human immunodeficiency viruses (Asres and Bucar, 2005; Lubbe et al., 2012), dengue virus, rotavirus, Zika virus (Oliveira et al., 2017; Akram et al., 2018), and SARS coronavirus (Prasad et al., 2020; Tsai et al., 2020; Yang et al., 2020). Different computational studies on the antiviral promises of herbal plants indicated that secondary metabolites present in plant extracts could interact with different targets of the SARS-CoV-2 virus. Table 1 summarizes some of the recently published plant-based secondary metabolites that have shown some therapeutic promises against SARS-CoV-2 in computational studies.

**TABLE 1:** Plant secondary metabolites as probable drug candidates for COVID-19.

Metabolites	Drug targets	Applied computational approach	Study group
Curcumin, nimbin, withaferin A, piperine, mangiferin, thebaine, berberine, and andrographolide	ACE-2 and spike protein	_	Maurya et al. (2020)
Chebulagic acid	ACE-2 and spike protein	Molecular docking	Krishnasamy et al. (2020)
Emodin, hesperidin, and chrysin	ACE-2 and spike protein	Molecular docking and modeling of protein	Basu et al. (2020)
Curcumin, epigallocatechin gallate, chrysophanol, and apigenin	Spike protein	Molecular docking	Subbaiyan et al. (2020)
Quercetin, magnoflorine, luteolin, tinosponone, cirsimaritin, chrysoeriol, and vasicinone	Spike protein	Molecular docking	Kiran et al. (2020)
Digitoxigenin, crocin, and *β*-eudesmol	Main protease	Molecular docking	Aanouz et al. (2020)
Folic acid, hispidin, and lepidine-E	Main protease	Structure activity relationship and molecular docking	Serseg et al. (2020)
Baicalin, hypericin, 3-glucoside, cyanidin, and glabridin	Main protease	_	Islam et al. (2020)
Benzylidenechromanones	Main protease	Molecular docking and bioinformatics	Sepay et al. (2020)
Leucoefdin	Main protease	Molecular docking and molecular dynamic simulation	Singh and Mishra (2020)
Arjunglucoside-I, carnosol, and rosmanol	Main protease	Virtual screening, molecular docking, and molecular dynamic simulation	Umesh et al. (2020)
Withaferin A, silybin, quercetin, cordioside, and catechin	Spike protein, MPro, and RdRp	Molecular docking	Pandit and Latha (2020)
(±) 6-acetonyldihydrochelerythrine, allocryptopine, and protopine	RdRp	Molecular docking	Pandeya et al. (2020)
Piceatannol, resveratrol, pinosylvin, and pterostilbene	ACE-2 receptor	Molecular docking	Wahedi et al. (2020)
Chloroquine, isothymol, and captopril	ACE-2 receptor	Drug-likeness, PASS and P450 site of metabolism prediction, pharmacophore mapper, molecular docking, and dynamic simulation	Abdelli et al. (2020)
Macaflavanone E, belachinal, and vibsanol B	Envelope protein	Protein sequence analysis, dynamic simulation, molecular docking, and intermolecular interaction	Gupta et al. (2020)
Myricitrin, amaranthin, calceolarioside B, licoleafol, methyl rosmarinat, and colistin	3CLpro	_	Rothan and Byrareddy (2020)
Cryptoquindoline, 10-hydroxyusambarensine, 20-epibryonolic acid, 22-hydroxyhopan-3-one, cryptospirolepine, 6-oxoisoiguesterin, and isoiguesterin	3CLpro	Drug-likeness, prediction analysis, and ligand–protein interaction	Gyebi et al. (2020)
Coumarine and flavone	3CLpro	Structure-based virtual screening and molecular dynamic simulation	Khan et al. (2020)
Nimocin, nimbolin A, and cycloartanols	Envelope and membrane proteins	Virtual screening, molecular dynamic simulation, and docking	Borkotoky and Banerjee (2020)
Glabridin, apigenin, glycoumarin, glucobrassicin, and oleanolic acid	6LU7 and 6Y2E proteases	Molecular docking analysis	Sampangi-Ramaiah et al. (2020)

Due to homology in SARS-CoV and SARS-CoV-2, the previous studies may also endorse the potential of naturally occurring compounds to inhibit SARS-CoV-2. Various studies are currently using traditional medicines and extracting their therapeutic potential against SARS-CoV-2 (Ang et al., 2020; Ren et al., 2020; Silveira et al., 2020). A well-studied traditional Chinese medicine (TCM) is reported to treat SARS-CoV and various other diseases (Tong et al., 2004; Cao et al., 2020; Huang et al., 2020; Zhang Q. et al., 2020; Zhang et al., 2021). This medicine is also applied to treat the patients of SARS-CoV-2, with approximately 85% success (Yang et al., 2020). The plants used in TCM contain active biomolecules like flavonoids (herbacetin, isobavaschacone, rhoifolin, quercetin 3-b-D-glucoside, epigallocatechin gallate, and pectolinarin) that regulate virus accumulation by restricting the multiplication. In a recent report, Sehailia and Chemat (2020) studied the infection mechanism of SARS-CoV-2, compared it with that of plasmodium, and reported that each pathogen causes lung infection by crystallizing carbon dioxide. Based on this finding, artemisinin, isolated from *Artemisia annua* L., has been proposed to treat SARS-CoV-2–infected patients. Artemisinin is commonly used to treat malaria and against various viral infections like MERS-CoV and SARS-CoV (D’Alessandro et al., 2020; Hahn et al., 2018). Collaborative research between the Max-Plank Research Center, Germany, and Mateon Therapeutics, California, is being performed using *Artemisia annua* L. to treat COVID-19–infected patients. This research is supported by the World Health Organization (WHO).

3-Chymotrypsin–like protease (3CLpro) is vital for replicating the virus, and thus represents a promising drug target for the development of therapeutic agents for SARS-CoV and other human coronaviruses, including SARS-CoV-2 (Yang et al., 2020). The following reports suggest that Chinese rhubarb extracts (Luo et al., 2009), *Houttuynia cordata* (Lau et al., 2008; Fung et al., 2011), flavonoids (Jo et al., 2019; Jo et al., 2020), beta-sitosterol extracted from the root extract of *Isatis indigotica*, and herbal extracts of TCM could inhibit the enzymatic activity of SARS 3CLpro. Besides that, the flavonoids, namely, herbacetin, isobavaschalcone, quercetin 3-β-D-glucoside, and helichrysetin, had shown their capability to block the enzymatic activity of MERS-CoV 3CL protease (Jo et al., 2019). The RNA-dependent RNA polymerase (RdRp) is a key enzyme responsible for both positive- and negative-strand RNA synthesis, and it is another potential target for a drug. It was reported by Fung et al. (2011) that the extracts of Kang Du Bu Fei Tang, *Sinomenium acutum*, *Coriolus versicolor*, and *Ganoderma lucidum*, inhibited SARS-CoV RdRp when tested in different doses (Fung et al., 2011).

### Essential Oils

The applications of essential oils, extracted from various medicinal plants like *Citrus* spp, *Mentha* spp, ginger, *Hyssopus officinalis* L., *Illicium* spp, and *Santalum* spp with antiviral effects, have been well studied by numerous researchers (Li and Peng, 2013; Akram et al., 2018; Dhama et al., 2018; Wink 2020). Ben-Shabat and coworkers reported that these essential oils alter the viral-envelope lipid-bilayer membrane's fluidity (Ben-Shabat et al., 2020). In 2003, Schnitzler reported that monoterpenes, oxygenated sesquiterpenes, and phenylpropanoids of essential oils could disrupt the phospholipid bilayer membrane of human coronavirus that interferes with the envelope protein structure of the virus during infection (Schnitzler et al., 2008).

A major study showed that eucalyptol (essential oil from gum trees, *Eucalyptus* spp.) is effective against SARS-CoV-2 and other coronaviruses because its major component, eucalyptus oil, consists of ketone, ether, and hydroxyl groups that play a crucial role in the inhibition of SARS-CoV-2 (Sharma, 2020). Another compound named jensenone, obtained from eucalyptus essential oil, has also shown antiviral potential to inhibit Mpro of COVID-19 (Sharma and Kaur, 2020).

### Alkaloids

Chloroquine is an alkaloid that is obtained from the cinchona tree’s bark. It has DNA intercalating properties and is identified as a potential candidate for developing an effective drug for SARS-CoV-2 (Devaux et al., 2020). Another alkaloid, Resochin, used in malaria treatment, has been thought to be an effective antiviral compound as it can interfere with the replication, transcription, and protein synthesis of viral RNA (Wink, 2020). The isoquinolines, for example, palmatine and chelidonine, are also promising biomolecules that could be potential drug candidates against COVID-19 (Ho et al., 2019; Wink, 2020).

Kim et al. (2019) studied the important bis-benzylisoquinoline alkaloids extracted from *Stephania tetrandra* S. Moore and related species of Menispermaceae, such as cepharanthine (CEP), tetrandrine (TET), and fangchinoline (OFAN). They investigated the antiviral activity of these alkaloids against HCoV-OC43 in human coronavirus–infected MRC-5 human lung cells (Kim et al., 2019). The result showed that all three of these alkaloids could decrease the replication of HCoV-OC43 inside host cells. Apart from that, they also inhibited the viral spike and nucleocapsid protein expression.

### Flavonoids, Phenolics, and Polyphenols

Flavonoids, phenolic compounds, steroids, polyphenols, and terpenoids, and their derivatives are commonly found in secondary metabolites of plants and consist of aromatic rings with one or many hydroxyl groups (Vieira et al., 2010; Wink, 2020). Wink's study revealed that polyphenols could bind with the lipoprotein of the virus envelope that checks the viral invasion in host cells. Various other studies confirmed the antiviral activity of phenolic compounds like curcumin, catechin, bavachinin, gallate, silvestrol, and tomentin (Ryu et al., 2010; Khaerunnisa et al., 2020; Wink, 2020; Yang et al., 2020). Besides phenolic compounds, flavonoids are also potential candidates against SARS 3CLpro enzymes of human coronavirus. Various antiviral studies on flavonoids have shown promising results (Table 2).

**TABLE 2:** List of the promising phytochemicals used worldwide for antiviral studies that could play a crucial role in the treatment of COVID-19.

Source (medical plants)	Antiviral compound(s)	Virus	Mode of antiviral effects	References
*Curcuma longa* L., *Camellia sinensis* (L.) Kuntze, *Mentha longifolia* (L.) L*., Phonix hanceana* var. loureiroi, *Capsicum annum* L*.,* and *Olea europea* L.	Glucoside, luteoloin-7, curcumin, de menthoxy curcumin, epicatechin- gallate, oleuropein, apigenin-7, and catechin	Coronavirus (CoV)	Mpro protein of COVID-19 was inhabited by all these antiviral compounds. However, further investigations are required.	Khaerunnisa et al. (2020)
*Tylophora indica* (Burm.f.) Mabb.	Tylophorine	CoV	These biomolecules showed broad-spectrum potential for inhibiting coronaviruses.	Yang et al. (2020)
*Lycoris radiata* (L'Hér.) Herb.	Lycorine	CoV	Lycorine could be a promising biomolecule for antiviral activity.	Suryanarayana and Banavath (2020)
*Psoralea corylifolia* (L.) Medik.	Bavachinin, corylifol, and psoralidin	CoV	The ethanol extract of these secondary metabolites showed potential activity against SARS-CoV PLpro.	Kim et al. (2014)
*Clivia miniata* (L.) Medik.	Mycophenolate mofetil and lycorine	HCov-OC43, MHV- A59, HCoV-NL63, and MERS-CoV	Mycophenolate mofetil demonstrated immune-suppressing effects on CoV, while lycorine showed inhibition of RNA, DNA, and protein synthesis that affects cell division.	Shen et al. (2019)
*Carapichea ipecacuanha* (Brot.) L. Andersson	Emetine		Emetine showed strong antiviral activity by blocking entry of MERS-CoV.	
*Aglaia foveolata* Pannell	Silvestrol	HCoV-229E	Silvestrol demonstrated strong inhibition of cap-dependent viral mRNA translation.	Muller et al. (2018)
*Broussonetia papyrifera* (L.) L'Hér. ex Vent.	Kazinol A, Kazinol F, Kazinol B, Kazinol J	Papain-like and 3-chymotrypsin–like CoV cysteine proteases	These polyphenols showed inhibition against both CL and PL CoV proteases.	Park et al. (2017)
*Broussonetia papyrifera* (L.) L'Hér. ex Vent.	Polyphenols, for example, biphenyl propanoid and broussochalcone A and B	CoV cysteine proteases	All of these polyphenols could be potential biomolecules for developing anti-CoV drugs.	Park et al. (2017)
Peel extracts of *Citrus sinensis* L.*, Anthemis hyaline,* and *Nigella sativa* L.	Essential oils	CoV-infected HeLa- epithelial carcinoembryonic antigen	Reduces the virus loads by downregulation of tryptophan- operon (TRP- gene) of CoV.	Ulasli et al. (2014)
*Paulownia tomentosa* (Thunb.) Steud.	Tomentin	SARS-CoV	These granulated flavonoids inhibit the proteases of SARS-CoV.	Cho et al. (2013)
*Camellia sinensis* (L.) Kuntze	Catechins	SARS-CoV	During screening of various teas, catechins showed strong inhibition for N-protein of SARS-CoV.	Roh (2012)
*Aglaia perviridis* Hiern	Myricetin and scutellarein	SARS-CoV	This study showed its effect against ATPase activity that leads to inhibition of the helicase protein of SARS-CoV.	Yu et al. (2012)
*Pelargonium sidoides* DC.	Extract EPs^®^ 7630	Human coronavirus (HCoV)	EPs^®^ 7630 interferes with replication of various respiratory viruses such as HCoV.	Michaelis et al. (2011)
*Eucalyptus globus*	1,8-cineol	SARS-CoV-2	Translocation of NF-kB p65 to the nucleus is inhibited, which negatively affects NFkB-driven transcription.	Greiner et al. (2013)
*Curcuma longa* L.	Curcumin	SARS-CoV-2	Curcumin showed inhibition of the Notch1-GATA3 signaling pathway and averted the progress of allergic inflammation.	Chong et al. (2014)
*Papaver somniferum* L.	Codeine	SARS-CoV-2	Codeine is metabolized to morphine in the animal body. It produces an analgesic effect by interacting with muopoid receptors, which are available in the cells of the nervous system (central and peripheral).	Bhandari et al. (2011), Kodaira and Spector (1988)
	Thebaine			

In recent research on biomolecules, Letko et al. (2020) reported that SARS-CoV-2 associates with host cells using angiotensin-converting enzyme 2 (hACE2), that is, a host receptor. Therefore, plant-based biomolecules that can inhibit the interaction with this receptor could become an excellent pharmaceutical candidate to fight against SARS-CoV-2. For further analysis, molecular docking was performed with cannabinoids and different phytochemicals to establish these biomolecules’ binding positions with viral spike protein (S) (Ho et al., 2007; Tallei et al., 2020).

## Identification of Therapeutic Biomolecules of Plants Through the Multi-Omics Approach

**FIGURE 4:**
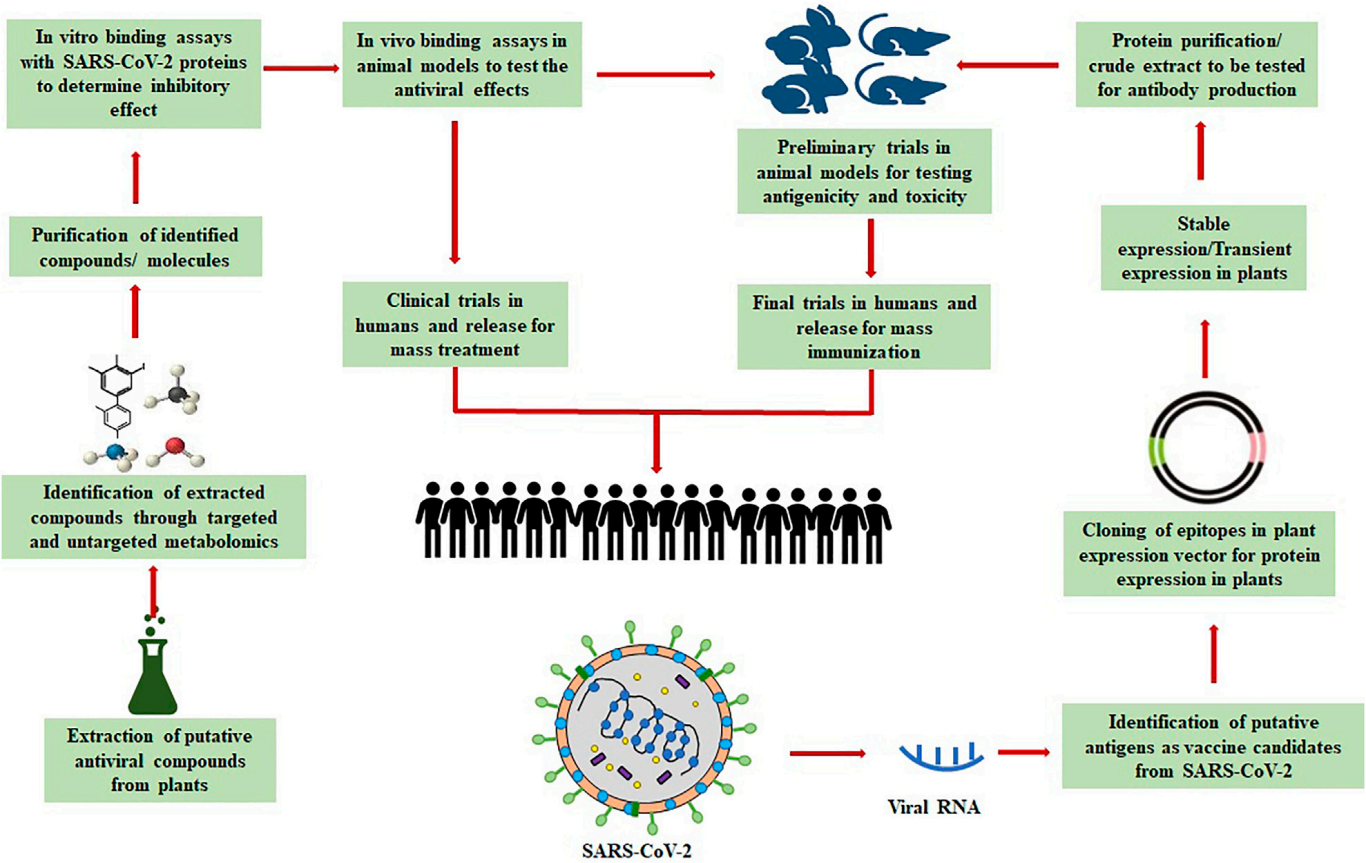
Schema representing steps to discover therapeutics (plant-based biomolecules and vaccines) for COVID-19. The biomolecules/plant metabolites can be screened and tested following the mentioned steps for potential antiviral activity against SARS-CoV-2. The figure also depicts the steps to develop vaccines by exploring the genetic material of the virus.

Before discussing the application of the multi-omics approach in the different research fields, it is essential to understand how all these technologies work individually and how information could be combined to generate a more in-depth understanding. Various methods can apply all the omics knowledge depending on the availability of data and requirements (Zhou et al., 2020; Zoppi et al., 2021). The data available in the public domain related to all these omics technologies are extracted for complex analysis, and attempts are made to link all markers at the different levels (genomic, proteomic, and metabolomic) back to annotated genes (Figure 4; Subramanian et al., 2020). Usually, this method works appropriately because well-curated and interpreted databases with a complete description of genes and their biological functions can be acquired from several data sources. However, it is a big challenge to collect all the information separately and make a common analysis. Multi-omics data are usually not cross-referenced between repositories. A web application, that is, MOD-Finder, searches for multi-omics datasets related to a user-defined chemical of interest (Canzler et al., 2020).

The commonly used and readily available databases are the gene database of NCBI (http://www.ncbi.nlm.nih.gov/gene), Gene Ontology (http://geneontology.org) (Carbon et al., 2019), Ensembl (http://useast.ensembl.org) (Andrew et al., 2020), KEGG (http://genome.jp/kegg/pathway.html), HMDB (http://hmdb.ca/), MetaCyc (http://metacyc.org/), Wikipathways (http://wikipathways.org/index.php/WikiPathways), and DAVID (http://david.abcc.ncifcrf.gov/P). Extensive work is underway on the genome using its different aspects. The forecasting or designating genotypes that cannot be assayed directly in individuals’ samples are known as genotype imputation (Ashburner et al., 2000; Naj, 2019; Chen S. F. et al., 2020). In recent years, the studies on genome-wide association (GWA) have revealed many significantly replicated associations for various complicated diseases (McGuire et al., 2020). In the analysis of GWA studies, genotype imputation has been widely used to enhance power, fine-map connections, and expedite the integration of results throughout the studies using meta-analysis. For genotype imputation, a resource like 1000 Genomes (http://www.1000genomes.org) (Marchini and Howie, 2010; Howie et al., 2011; Belsare et al., 2019) is available that facilitated combining various genotyping platforms and consequently enhanced the capability to interpret the genomics data and execute meta-analyses. Although some forms of data cannot be easily mapped to annotated genes, such limitations of annotation are addressed with the newest omics technologies.

Metabolomics is seen with considerable gaps in the annotation, restricting the efficacy of pathway-based and integrative method approaches (Kilk, 2020). Metabolomic datasets are mostly deciphered in the form of metabolic pathways. The KEGG database consists of metabolic pathways and contains information about both enzymes and metabolites. This database is categorized into groups related to cellular processes, metabolism, human diseases, *etc*. (Kanehisa and Sato, 2020). However, the annotated metabolites are very limited, suggesting that there is a lot to be learned regarding the role of several metabolites in human health.

Due to the availability and storage of the extensive amount of molecular data, an urgent demand has developed as a new branch of science: “system biology,” which unravels the basic functional properties of living beings originating from the interaction of macromolecules (Zupanic et al., 2020). The increased ability to elucidate genetic variations and their role in downstream molecular changes, like metabolite levels, would play a crucial role in interpreting and combining various data types.

Multi-omics approaches can be used to identify and screen plant-based biomolecules. They also provide deep insights into the effect of these biomolecules on COVID-19. The addition of proteomic datasets to genomic and transcriptomic data helps to understand the role of plant-based biomolecules against COVID-19. The integration of proteomics data complements genomics in the identification of multiple pathways. As soon as any cell is given exposure to biomolecules, it triggers a series of effects at the regulatory pathway level, which involve changes of levels and interactions of different types of biomolecules. Transcriptomics can detect biomolecules of one type and can only capture changes in a small subset of the biological cascade.

How to employ multi-omics data for the study is solely dependent on integration strategies. Various methods are available for the integrative analysis of multi-omics data. This review focuses on implementing these approaches for screening potential therapeutic biomolecules, which can be efficiently used against COVID-19. The comprehensive reviews on multi-omics integration methods are provided by various researchers (Cavill et al., 2016; Huang et al., 2017; Tarazona et al., 2018; Subramanian et al., 2020).

### Genomics Approaches for Production of Plant-Based Biomolecules

Plant biotechnology provides a comprehensive platform with several strategies to facilitate the synthesis of biomolecules on a large scale, viz. hairy root culture, cell suspension culture, *etc*. The advancement of genomics can accelerate the gene manipulation and pathway triggering the biosynthesis of therapeutically active compounds. Genetic manipulation of genes and genomes in plants can be achieved by various methods, including transformation (development of transgenic), inducible and transient expression systems, gene-silencing methodologies, knockout, knockdown, and the most advanced genome-editing (Wang et al., 2020c). In pandemic situations, the best vaccine production method is transient expression because it is time-saving and could be advanced for large-scale manufacturing. The most advanced next-generation sequencing offers detailed information about the genes and complete genomes, including the noncoding region and regulatory elements that facilitate the identification of genes associated with biomolecules’ biosynthesis (Peška et al., 2017). Gene cloning helps in the identification and molecular characterization of the genes that encode biomolecules. Furthermore, it can be transformed into a plant system for the expression and optimization of the product. For example, *Nicotiana benthamiana* Domin extends a systematic and effective system and is used to express VLP of SARS-CoV-2 to produce the plant-based vaccine (Rattanapisit et al., 2020). This system could yield the required biomolecules that can be scaled up, isolated, and purified by downstream approaches (Sharma N. et al., 2020).

The CRISPER/Cas9 (genome editing) approach facilitates accurate editing of genes (Dangi et al., 2019). Specifically, this can be applied to knock out the enzymes that involve the target biomolecule as a precursor. It accelerates the processes with the overproduction of required metabolites. The higher level of biomolecule production and fine-tuning of biosynthetic machinery could be attained by using transient methods like virus-induced gene silencing or RNA interference. A genetic transformation like *Agrobacterium*-mediated transformation provides the stable and reliable change and expression of genes in the plants (Ma et al., 2020). This method could easily be customized to develop target biomolecules in different plants, which can further undergo downstream processing and convenient purifications. Some of the useful phytochemicals are found in endangered species viz. Chinese medicinal plants *Panax ginseng* C. A. Mey. and *Magnolia officinalis* Rehder and E. H. Wilson, but their use in therapeutics is prohibited. Genetic manipulation and transformation methods offer model plant species as an alternative. The biosynthetic pathway can be studied and engineered into these, such as tomato, tobacco, rice, and maize. This has the advantage of producing beneficial compounds without putting any pressure on the original medicinal plant (Sassi et al., 2008; Moon et al., 2019). An excellent example of the application of genomics and biotechnology is the saponin glycyrrhizin. It is a naturally occurring class of compounds and is reported to inhibit coronavirus replication, and its antiviral activity augments ten folds by modification in its glycosidic chain (Hoever et al., 2005). The successful whole nucleic acid sequencing of SARS-CoV-2 from a different population of patients has given a new vision regarding the pathogen and its nonuniformity worldwide. As sequence data on the virus’s proteomics and metabolomics are available, they could help study it more precisely (Gordon et al., 2020; Shen et al., 2020; Wang M. et al., 2020).

### Deploying Proteomics Approaches for Plant-Based Biomolecules and Their Interaction with COVID-19

Proteomics is the most powerful tool for studying total expressed proteins in an organism or cell type at a particular time. This provides the methodologies used for identification, detection, sample preparation, separation methods, and quantification of proteins. Proteins are responsible for the cells’ function, and the expression, localization, and activity of proteins differ in various conditions. Hence, the study of protein expression in cell types or different conditions helps to identify and understand their biological information. All plant biomolecules derive from specific biosynthetic pathways. The comprehensive study of those pathways, starting from the analysis of the genes underlying them and biosynthetic enzymes and their regulation (Song et al., 2015), is another challenging task that can be achieved by deploying different proteomics methods. Mass spectrometry (MS) is an important technique that enables the analysis of proteomes and identification of proteins present in the biological system. The separation of proteomes can be performed by gel chromatography or liquid chromatography before analysis. Production of allergens and toxins while deriving a specific plant biomolecule should also be monitored to check if any toxic by-product is also produced. All such allergens and toxins are identified and systematized in www.allergenonline.org/ and www.allergen.org (Ahsan et al., 2016; Croote and Quake, 2016). Identification of accurate therapeutic plant biomolecules is the critical and key step. Once they are identified, their interaction and effect on cells and cellular activities in another aspect of proteomics and proteomics research can help identify medicinal plant biomolecules.

Proteomics relates proteins’ functional role to host and pathogen (Zheng and Perlman, 2018). Zheng and Perlman (2018) studied proteomics’ role in the host immune system and its responses to the respiratory virus interactions. Other researchers also studied G-protein–coupled receptors (Sriram and Insel, 2018), enzymes (Ding et al., 2017), and ion channels (Duncan et al., 2020). Recently, various protein-based analyses of coronavirus have been done to identify structural proteins that include SDS-PAGE analysis, Western blot, protein categorization, protein identification, separation, and quantification. The proteome microarray of SARS-CoV-2 was demonstrated by Wang et al., which helps in mapping COVID-19 antibody interactions (Wang X. et al., 2020). A detailed report was given by Gordon et al. on the human protein–protein interaction map of SARS-CoV-2, where they defined 332 human protein–protein interactions, and out of them, 66 were targeted by several preexisting FDA-approved drugs or under trial drugs (Gordon et al., 2020).

### Transcriptomics and Metabolomic Approaches

Transcriptomics studies primarily deal with gene expression profiles, that is, by RNA sequencing (Depledge et al., 2019), ribosome profiling, and high-throughput DNA microarray studies (Wang et al., 2009). Transcriptomics is an approach for exhaustive study and detection of RNA in the cell. In transcriptomics, a pathway response is mainly detected via a known set of target genes of the pathway expressed differentially. The information on association with a particular pathway is mainly available for protein-coding RNAs (mRNAs). Earlier, transcriptomics was dependent on microarrays as a measurement technique. Microarrays constitute a targeted detection approach; that is, they require prior selection and knowledge of the sequence of the interrogated RNAs. Recently, transcriptomics has switched to transcriptome sequencing (RNA-seq), which provides a platform for simultaneous identification of transcripts, isoform detection, and quantification (Canzler et al., 2020). The dose–response models were generated to determine the factors affecting gene expressions (Hashem et al., 2019) at various viral protein concentrations (Haas, 2020). Studies were also conducted to check the concentration of mRNA at different infection stages and its propagation (Albarino et al., 2018).

The metabolome is a collection of chemically highly heterogeneous molecules. It can be defined as the complete complement of all small molecule metabolites found in a specific cell, organ, or organism (Wishart, 2007). Different metabolic enzymes run cellular metabolism (Ahmed et al., 2020), and this is reported by a fierce study of genomics, proteomics, and transcriptomics (Fanos et al., 2020). These are directly connected with pathways available in metabolomics (Haas et al., 2016). The primary importance of metabolomics is related to diagnostics (Debnath et al., 2010; To et al., 2016). The concentration of metabolites is observed and identified by high-performance liquid chromatography/mass spectrometry (HPLC/MS) and nuclear magnetic resonance (NMR) (Peng and Liu, 2017). The early studies concluded that metabolic profile analysis reveals the inactivation and binding of metabolites with the therapeutic compounds (Eisfeld et al., 2017).

### Applications of Artificial Intelligence in Multi-Omics Data Analysis

**FIGURE 5:**
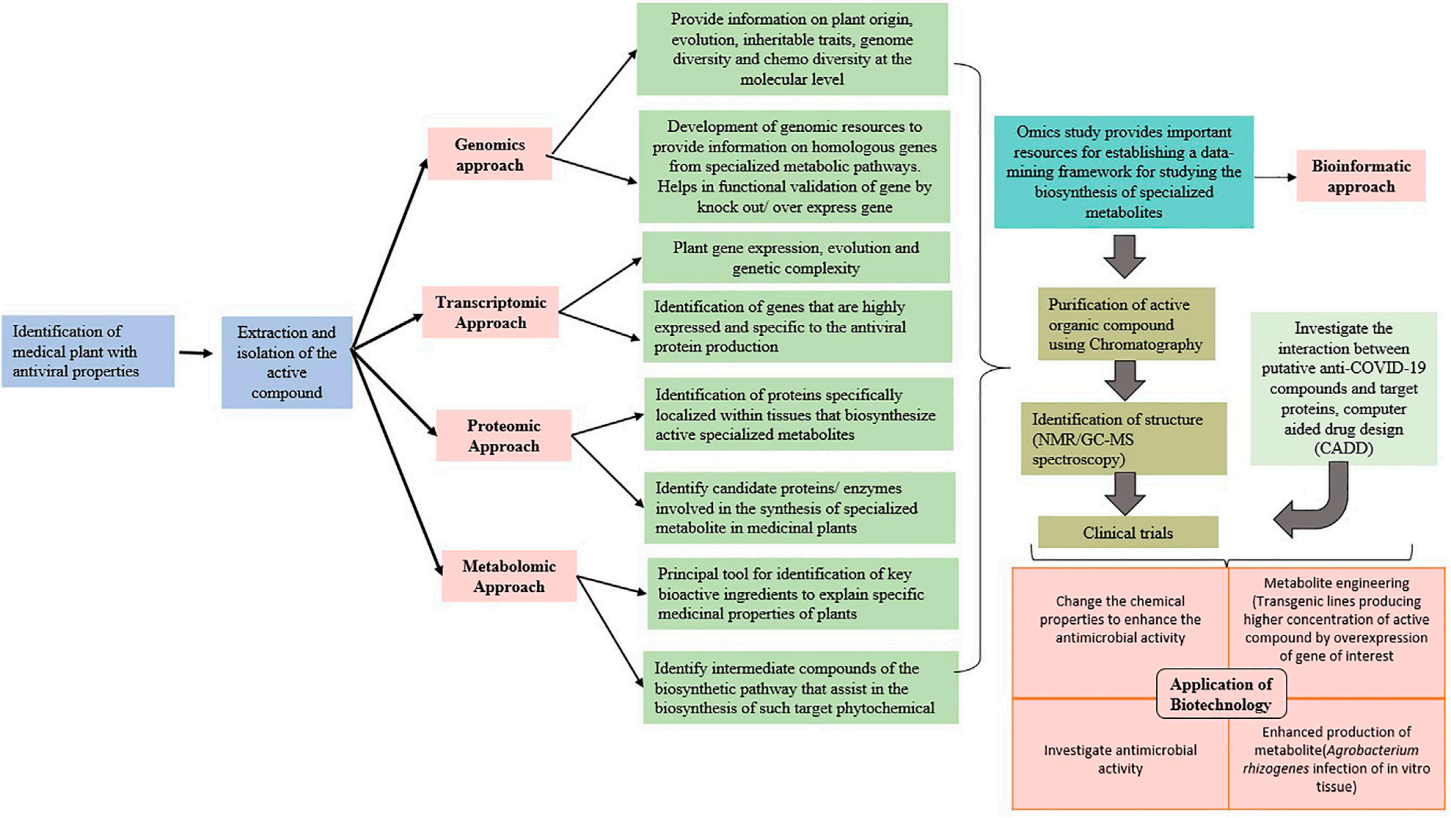
Use of biotechnology and multi-omics approaches for screening and profiling of plant-based biomolecules against SARS-CoV-2.

Artificial intelligence (AI) is the computational design, development, and application of computer programs and algorithms that perform cognitive functions based on human intelligence traits, for example, anticipating, problem-solving, and learning (Saxena et al., 2019; Sharma A. et al., 2020). AI techniques have the potential to accelerate the virtual screening, lead discovery and validation, *etc*. (Kumar et al., 2011; Kumar et al., 2017), thereby assisting in drug design and repurposing, and they can complement the traditional drug development methods for COVID-19. AI technique–based Benevolent AI has been successfully applied to identify baricitinib as a potential drug against COVID-19 (Favalli et al., 2020; Randhawa et al., 2020). PolypharmDB (Redka et al., 2020) and inclProject IDentif.AI (Abdulla et al., 2020) have already been successful in identifying potential drug candidates against SARS-Cov-2. A supervised learning–based Vaxign reverse vaccinology-machine learning platform has been developed for assisting the development of vaccine candidates against COVID-19 (Ong et al., 2020). Figure 5 summarizes the applications of biotechnology and multi-omics approaches for screening and profiling of plant-based biomolecules against SARS-CoV-2.

Wang et al. (2020d) developed a deep learning and ontology-based side effect prediction framework to evaluate and assess traditional Chinese medicines against COVID-19 treatment. Moreover, AI techniques are efficiently applied in SARS-CoV-2 protein structure prediction (Zhang et al., 2004; Senior et al., 2020). Apart from assisting the drug design, drug repurposing, and vaccine candidate development, AI has also been instrumental in spreading awareness, curbing misinformation (Hung et al., 2020; Miner et al., 2020; Rashid and Wang, 2020), assisting in early diagnosis (Xu et al., 2020), and decreasing the burden in healthcare professions by providing accurate decision support (Iwendi et al., 2020) during the COVID-19 pandemic.

## Conclusion

The present review gives an insight into the applicability of multi-omics tools and different omics approaches in identifying potential therapeutic plant biomolecules. These tools could explore the immense potential of plant-based biomolecules for the prevention, mitigation, or cure of SARS-CoV-2–infected patients. There is a need to reduce the gaps between the conventional treatment from plant extracts and herbs with an updated understanding of biomolecules/phytochemicals present in plant extracts using omics technologies. Advancement in technology and discovery of different omics approaches to explore and analyze the genomic, proteomic, and metabolomic data can generate the profile of plant biomolecules and identify the potential antiviral compounds that could be used against SARS-CoV-2.

Due to the highly contagious nature of SARS-CoV-2, handling of clinical samples in omics research facilities is often restricted. This has made the implementation of system-level molecular research extremely challenging. With this limitation, it is helpful for academicians, scientists, and health professionals from this field to be aware of the recent trends in omics approaches to address issues related to COVID-19. Various plant-based biomolecules have already been identified and studied at different levels of omics research. Now, screening those potential therapeutic compounds to treat SAR-CoV-2–infected patients and devising a relevant strategy to optimize the production and purification of those biomolecules using multi-omics approaches are the urgent needs of the situation. Multi-omics techniques are anticipated to play a crucial role in the identification of potential therapeutic plant biomolecules and effective clinical management of COVID-19. Once these approaches have been applied successfully, the screened repository of plant-based therapeutic biomolecules could be used for future health emergencies like the emergence of a new strain or mutation in the virus. We hope that this document may help future researchers to quickly get an overview and understand the applications of omics approaches to find out therapeutically active plant-based biomolecules in infectious outbreaks or pandemics.

## References

[B1] Aanouz I. Belhassan A. El-Khatabi K. Lakhlifi T. El-ldrissi M. Bouachrine M. (2020). Moroccan Medicinal Plants as Inhibitors against SARS-CoV-2 Main Protease: Computational Investigations. J. Biomol. Struct. Dyn. 2020, 1–9. 10.1080/07391102.2020.1758790 PMC7212546 32306860

[B2] Aaron G. Daire C. Leticia M. W. Simon J. W. (2020). Multi-Omics Technologies Applied to Tuberculosis Drug Discovery. Appl. Sci. 10, 4629. 10.3390/app10134629

[B3] Abdelli I. Hassani F. Bekkel Brikci S. Ghalem S. (2020). In Silico study the Inhibition of Angiotensin Converting Enzyme 2 Receptor of COVID-19 by Ammoides Verticillata Components Harvested from Western Algeria. J. Biomol. Struct. Dyn. 2020, 1–14. 10.1080/07391102.2020.1763199 PMC7232889 32362217

[B4] Abdulla A. Wang B. Qian F. Kee T. Blasiak A. Ong Y. H. (2020). Project IDentif.AI: Harnessing Artificial Intelligence to Rapidly Optimize Combination Therapy Development for Infectious Disease Intervention. Adv. Therap. 3, 2000034. 10.1002/adtp.202000034 PMC7235487 32838027

[B5] Ahmed I. Hyder S. Altayb N. (2020). The Important Role of Fever during Infection and the Use of Fever for Treatment of Coronavirus Patients. SSRN Elec J. 10.2139/ssrn.3556215

[B6] Ahsan N. Rao R. S. P. Gruppuso P. A. Ramratnam B. Salomon A. R. (2016). Targeted Proteomics: Current Status and Future Perspectives for Quantification of Food Allergens. J. Proteomics 143, 15–23. 10.1016/j.jprot.2016.04.018 27113134 PMC5082697

[B7] Akram M. Tahir I. M. Shah S. M. A. Mahmood Z. Altaf A. Ahmad K. (2018). Antiviral Potential of Medicinal Plants against HIV, HSV, Influenza, Hepatitis, and Coxsackievirus: a Systematic Review. Phytotherapy Res. 32, 811–822. 10.1002/ptr.6024 29356205

[B8] Al-Ishaq R. K. Overy A. J. Büsselberg D. (2020). Phytochemicals and Gastrointestinal *Cancer*: Cellular Mechanisms and Effects to Change *Cancer* Progression. Biomolecules 10 (1), 105. 10.3390/biom10010105 PMC7022462 31936288

[B9] Al-Jabir K. Insel P. A. (2018). G Protein-Coupled Receptors as Targets for Approved Drugs: How Many Targets and How Many Drugs?. Mol. Pharmacol. 93, 251–258. 10.1124/mol.117.111062 29298813 PMC5820538

[B10] Albarino C. G. Guerrero L. W. Chakrabarti A. K. Nichol S. T. (2018). Transcriptional Analysis of Viral mRNAs Reveals Common Transcription Patterns in Cells Infected by Five Different Filoviruses. PLoS One 13, e0201827. 10.1371/journal.pone.0201827 30071116 PMC6072132

[B11] Anand A. V. Balamuralikrishnan B. Kaviya M. Bharathi K. Parithathvi A. Arun M. (2021). Medicinal Plants, Phytochemicals, and Herbs to Combat Viral Pathogens Including SARS-CoV-2. Molecules 26 (6), 1775. 10.3390/molecules26061775 33809963 PMC8004635

[B12] Andrew D. Y. Premanand A. Wasiu A. James A. Jamie A. Jorge A. (2020). Ensembl 2020. Nucleic Acids Res. 48, D682–D688. 10.1093/nar/gkz966 31691826 PMC7145704

[B13] Ang L. Song E. Lee H. W. Lee M. S. (2020). Herbal Medicine for the Treatment of Coronavirus Disease 2019 (COVID-19): A Systematic Review and Meta-Analysis of Randomized Controlled Trials. J Clin Med. 9 (5), 1583. 10.3390/jcm9051583 PMC7290825 32456123

[B14] Arbab A. H. Parvez M. K. Al-Dosari M. S. Al-Rehaily A. J. (2017). *In vitro* evaluation of Novel Antiviral Activities of 60 Medicinal Plants Extracts against Hepatitis B Virus. Exp. Ther. Med 14, 626–634. 10.3892/etm.2017.4530 28672977 PMC5488430

[B15] Ashburner M. Ball C. A. Blake J. A. Botstein D. Butler H. Cherry J. M. (2000). Gene Ontology: Tool for the Unification of Biology. Nat. Genet. 25, 25–29. 10.1038/75556 10802651 PMC3037419

[B16] Asres K. Bucar F. (2005). Anti-HIV Activity against Immunodeficiency Virus Type 1 (HIV-I) and Type II (HIV-II) of Compounds Isolated from the Stem Bark of *Combretum Molle* . Ethiop Med. J. 43, 15–20. 16370525

[B17] Balkrishna A. Haldar S. Singh H. Roy P. Varshney A. (2021). Coronil, a Tri-herbal Formulation, Attenuates Spike-Protein-Mediated SARS-CoV-2 Viral Entry into Human Alveolar Epithelial Cells and Pro-Inflammatory Cytokines Production by Inhibiting Spike Protein-ACE-2 Interaction. J. Inflamm. Res. 14, 869–884. 10.2147/JIR.S298242 33758527 PMC7981146

[B18] Basu A. Sarkar A. Maulik U. (2020). Computational Approach for the Design of Potential Spike Protein Binding Natural Compounds in SARS- CoV2. Pharmacodynamics. 10.21203/rs.3.rs-33181/v1

[B19] Behl T. Kaur I. Bungau S. Kumar A. Uddin M. S. Kumar C. (2020). The Dual Impact of ACE2 in COVID-19 and Ironical Actions in Geriatrics and Pediatrics with Possible Therapeutic Solutions. Life Sci. 257, 118075. 10.1016/j.lfs.2020.118075 32653522 PMC7347488

[B20] Belfiore M. P. Urraro F. Grassi R. Giacobbe G. Patelli G. Cappabianca S. (2020). Artificial Intelligence to Codify Lung CT in Covid-19 Patients. Radiol. Med. 125 (5), 500–504. 10.1007/s11547-020-01195-x 32367319 PMC7197034

[B21] Belsare S. Levy-Sakin M. Mostovoy Y. Durinck S. Chaudhuri S. Xiao M. (2019). Evaluating the Quality of the 1000 Genomes Project Data. BMC genomics 20 (1), 620. 10.1186/s12864-019-5957-x 31416423 PMC6696682

[B22] Ben-Shabat S. Yarmolinsky L. Porat D. Dahan A. (2020). Antiviral Effect of Phytochemicals from Medicinal Plants: Applications and Drug Delivery Strategies. Drug Deliv. Transl. Res. 10, 354–367. 10.1007/s13346-019-00691-6 31788762 PMC7097340

[B23] Bhandari M. Bhandari A. Bhandari A. (2011). Recent Updates on Codeine. Pharm. Methods 2, 3–8. 10.4103/2229-4708.81082 23781422 PMC3658028

[B24] Bojkova D. Klann K. Koch B. Widera M. Krause D. Ciesek S. (2020). Proteomics of SARS-CoV-2-Infected Host Cells Reveals Therapy Targets. Nature 583 (7816), 469–472. 10.1038/s41586-020-2332-7 32408336 PMC7616921

[B25] Borkotoky S. Banerjee M. (2020). A Computational Prediction of SARS-CoV-2 Structural Protein Inhibitors from Azadirachta indica (Neem). J. Biomol. Struct. Dyn. 2020, 1–11. 10.1080/07391102.2020.1774419 PMC7311162 32462988

[B26] Canzler S. Schor J. Busch W. Schubert K. Rolle-Kampczyk U. E. Seitz H. (2020). Prospects and Challenges of Multi-Omics Data Integration in Toxicology. Arch. Toxicol. 94, 371–388. 10.1007/s00204-020-02656-y 32034435

[B27] Cao P. Wu S. Wu T. Deng Y. Zhang Q. Wang K. (2020). The Important Role of Polysaccharides from a Traditional Chinese Medicine-Lung Cleansing and Detoxifying Decoction against the COVID-19 Pandemic. Carbohydr. Polym. 240, 116346. 10.1016/j.carbpol.2020.116346 32475597 PMC7175912

[B28] Carbon S. Douglass E. Dunn N. Good B. Harris N. L. Lewis S. E. (2019). The Gene Ontology Resource: 20 Years and Still GOing Strong. Nucleic Acids Res. 47 (D1), D330–D338. 10.1093/nar/gky1055 30395331 PMC6323945

[B195] Cavill R. Jennen D. Kleinjans J. Briedé J. J. (2016). Transcriptomic and Metabolomic Data Integration. Br. Bioinform. 17 (5), 891–901. 10.1093/bib/bbv090 26467821

[B29] Chantrill B. H. Coulthard C. E. Dickinson L. Inkley G. W. Morris W. Pyle A. H. (1952). The Action of Plant Extracts on a Bacteriophage of *Pseudomonas* Pyocyanea and on Influenza A Virus. J. Gen. Microbiol. 6, 74–84. 10.1099/00221287-6-1-2-74 14927853

[B30] Chen B. Garmire L. Calvisi D. F. Chua M.-S. Kelley R. K. Chen X. (2020). Harnessing Big 'omics' Data and AI for Drug Discovery in Hepatocellular Carcinoma. Nat. Rev. Gastroenterol. Hepatol. 17, 238–251. 10.1038/s41575-019-0240-9 31900465 PMC7401304

[B31] Chen S. F. Dias R. Evans D. Salfati E. L. Liu S. Wineinger N. E. (2020). Genotype Imputation and Variability in Polygenic Risk Score Estimation. Genome Med. 12 (1), 100. 10.1186/s13073-020-00801-x 33225976 PMC7682022

[B32] Cho J. K. Curtis-Long M. J. Lee K. H. Kim D. W. Ryu H. W. Yuk H. J. (2013). Geranylated Flavonoids Displaying SARS-CoV Papain-like Protease Inhibition from the Fruits of *Paulownia Tomentosa* . Bioorg. Med. Chem. 21, 3051–3057. 10.1016/j.bmc.2013.03.027 23623680 PMC7126831

[B33] Chojnacka K. Witek-Krowiak A. Skrzypczak D. Mikula K. Młynarz P. (2020). Phytochemicals Containing Biologically Active Polyphenols as an Effective Agent against Covid-19-Inducing Coronavirus. J. Funct. Foods 73, 104146. 10.1016/j.jff.2020.104146 32834835 PMC7392194

[B34] Chong L. Zhang W. Nie Y. Yu G. Liu L. Lin L. (2014). Protective Effect of Curcumin on Acute Airway Inflammation of Allergic Asthma in Mice through Notch1-GATA3 Signaling Pathway. Inflammation 37, 1476–1485. 10.1007/s10753-014-9873-6 24706026 PMC4174331

[B35] Clark L. K. Green T. J. Petit C. M. (2021). Structure of Nonstructural Protein 1 from SARS-CoV-2. J. Virol. 95 (4), e02019–20. 10.1128/JVI.02019-20 33234675 PMC7851544

[B36] Croote D. Quake S. R. (2016). Food Allergen Detection by Mass Spectrometry: the Role of Systems Biology. Npj Syst. Biol. Appl. 2, 16022. 10.1038/npjsba.2016.22 28725476 PMC5516885

[B37] D’Alessandro S. Scaccabarozzi D. Signorini L. Perego F. Ilboudo D. P. Ferrante P. (2020). The Use of Antimalarial Drugs against Viral Infection. Microorganisms 8, 85. 10.3390/microorganisms8010085 PMC7022795 31936284

[B38] Dangi A. Yu S. Luo X. (2019). Emerging Approaches and Technologies in Transplantation: the Potential Game Changers. Cell Mol Immunol 16 (4), 334–342. 10.1038/s41423-019-0207-3 30760918 PMC6462010

[B39] Debiaggi M. Pagani L. Cereda P. M. Landini P. Romero E. (1988). Antiviral Activity of *Chamaecyparis Lawsoniana* Extract: Study with Herpes Simplex Virus Type 2. Microbiologica 11, 55–61. 2832710

[B40] Debnath M. Prasad G. B. K. S. Bisen P. S. (2010). Molecular Diagnostics: Promises and Possibilities Dordrech Heidelberg. London: Springer, 11–31.

[B41] Depledge D. P. Srinivas K. P. Sadaoka T. Bready D. Mori Y. Placantonakis D. G. (2019). Direct RNA Sequencing on Nanopore Arrays Redefines the Transcriptional Complexity of a Viral Pathogen. Nat. Commun. 10, 1–13. 10.1038/s41467-019-08734-9 30765700 PMC6376126

[B42] Devaux C. A. Rolain J.-M. Colson P. Raoult D. (2020). New Insights on the Antiviral Effects of Chloroquine against Coronavirus: what to Expect for COVID-19?. Int. J. Antimicrob. Agents 55, 105938. 10.1016/j.ijantimicag.2020.105938 32171740 PMC7118659

[B43] Dhama K. Karthik K. Khandia R. Munjal A. Tiwari R. Rana R. (2018). Medicinal and Therapeutic Potential of Herbs and Plant Metabolites/ Extracts Countering Viral Pathogens - Current Knowledge and Future Prospects. Curr. Drug Metab. 19, 236–263. 10.2174/1389200219666180129145252 29380697

[B44] Ding Y. Tang J. Guo F. (2017). Identification of Drug-Target Interactions via Multiple Information Integration. Inf. Sci. 418-419, 546–560. 10.1016/j.ins.2017.08.04510.1016/j.ins.2017.08.045

[B45] Drake K. B. Ganeshpurkar A. Mishra M. K. (2020). Natural RNA Dependent RNA Polymerase Inhibitors: Molecular Docking Studies of Some Biologically Active Alkaloids of Argemone Mexicana. Med. Hypotheses 144, 109905. 10.1016/j.mehy.2020.109905 32535456 PMC7262531

[B46] Duncan A. L. Song W. Sansom M. S. P. (2020). Lipid-dependent Regulation of Ion Channels and G Protein-Coupled Receptors: Insights from Structures and Simulations. Annu. Rev. Pharmacol. Toxicol. 60, 31–50. 10.1146/annurev-pharmtox-010919-023411 31506010

[B47] Eisfeld A. J. Halfmann P. J. Wendler J. P. Kyle J. E. Burnum-Johnson K. E. Peralta Z. (2017). Multi-platform 'Omics Analysis of Human Ebola Virus Disease Pathogenesis. Cell Host *&* Microbe 22, 817–829. 10.1016/j.chom.2017.10.011 29154144 PMC5730472

[B48] Enmozhi S. K. Raja K. Sebastine I. Joseph J. (2020). Andrographolide as a Potential Inhibitor of SARS-CoV-2 Main Protease: an In Silico Approach. J. Biomol. Struct. Dyn. 5, 1–7. 10.1080/07391102.2020.1760136 PMC7212536 32329419

[B49] Fanos V. Pintus M. C. Pintus R. Marcialis M. A. (2020). Lung Microbiota in the Acute Respiratory Disease: from Coronavirus to Metabolomics. J. Pediatr. Neonatal. Individual. Med. 9, e090139. 10.7363/090139

[B50] Favalli E. G. Biggioggero M. Maioli G. Caporali R. (2020). Baricitinib for COVID-19: a Suitable Treatment?. Lancet Infect. Dis. 20, 1012–1013. 10.1016/S1473-3099(20)30262-0 PMC7270794 32251638

[B51] Fung K. P. Leung P. C. Tsui K. W. Wan C. C. Wong K. B. Waye M. Y. (2011). Immunomodulatory Activities of the Herbal Formula Kwan Du Bu Fei Dang in Healthy Subjects: a Randomised, Double-Blind, Placebo-Controlled Study. Hong Kong Med. J. 17 (Suppl. 2), 41–43. 21368336

[B197] Gordon D. E. Jang G. M. Bouhaddou M. Xu J. Obernier K. White K. M. (2020). A SARS-CoV-2 Protein Interaction Map Reveals Targets for Drug Repurposing. Nature 583 (7816), 459–468. 10.1038/s41586-020-2286-9 32353859 PMC7431030

[B52] Greiner J. F.-W. Müller J. Zeuner M.-T. Hauser S. Seidel T. Klenke C. (2013). 1,8-Cineol Inhibits Nuclear Translocation of NF-κB P65 and NF-κB-dependent Transcriptional Activity. Biochim. Biophys. Acta. 1833, 2866–2878. 10.1016/j.bbamcr.2013.07.001 23872422

[B53] Gupta M. K. Vemula S. Donde R. Gouda G. Behera L. Vadde R. (2020). In-silico Approaches to Detect Inhibitors of the Human Severe Acute Respiratory Syndrome Coronavirus Envelope Protein Ion Channel. J. Biomol. Struct. Dyn. 39, 2617–2627. 10.1080/07391102.2020.1751300 32238078 PMC7171389

[B54] Gyebi G. A. Ogunro O. B. Adegunloye A. P. Ogunyemi O. M. Afolabi S. O. (2020). Potential Inhibitors of Coronavirus 3-chymotrypsin-like Protease (3CLpro): an In Silico Screening of Alkaloids and Terpenoids from African Medicinal Plants. J. Biomol. Struct. Dyn. 2020, 1–13. 10.1080/07391102.2020.1764868 PMC7256353 32367767

[B55] Haas C. T. Roe J. K. Pollara G. Mehta M. Noursadeghi M. (2016). Diagnostic ‘omics’ for Active Tuberculosis. BMC Med. 14, 37. 10.1186/s12916-016-0583-9 27005907 PMC4804573

[B56] Hahn F. Fröhlich T. Frank T. Bertzbach L. D. Kohrt S. Kaufer B. B. (2018). Artesunate-derived Monomeric, Dimeric and Trimeric Experimental Drugs - Their Unique Mechanistic Basis and Pronounced Antiherpesviral Activity. Antiviral Res. 152, 104–110. 10.1016/j.antiviral.2018.02.013 29458133

[B57] Hart O. E. Halden R. U. (2020). Computational Analysis of SARS-CoV-2/COVID-19 Surveillance by Wastewater-Based Epidemiology Locally and Globally: Feasibility, Economy, Opportunities and Challenges. Sci. Total Environ. 730, 138875. 10.1016/j.scitotenv.2020.138875 32371231 PMC7175865

[B58] Hasana S. Hossain M. F. Jalouli M. Kabir M. T. Uddin M. G. Wahed M. I. I. (2021). Genetic Diversity of SARS-CoV2 and Environmental Settings: Possible Association with Neurological Disorders. Mol. Neurobiol. 58, 1917–1931. Epub ahead of print. PMID: 33404977. 10.1007/s12035-020-02239-z 33404977 PMC7786338

[B59] Hashem A. M. Algaissi A. Agrawal A. S. Al-Amri S. S. Alhabbab R. Y. Sohrab S. S. (2019). A Highly Immunogenic, Protective, and Safe Adenovirus-Based Vaccine Expressing Middle East Respiratory Syndrome Coronavirus S1-CD40L Fusion Protein in a Transgenic Human Dipeptidyl Peptidase 4 Mouse Model. J. Infect. Dis. 220, 1558–1567. 10.1093/infdis/jiz13710.1093/infdis/jiz137 30911758 PMC7107499

[B60] Hillen H. S. Kokic G. Farnung L. Dienemann C. Tegunov D. Cramer P. (2020). Structure of Replicating SARS-CoV-2 Polymerase. Nature 584 (7819), 154–156. 10.1038/s41586-020-2368-8 32438371

[B61] Ho T. Wu S. Chen J. Li C. Hsiang C. (2007). Emodin Blocks the SARS Coronavirus Spike Protein and Angiotensin-Converting Enzyme 2 Interaction. Antiviral Res. 74, 92–101. 10.1016/j.antiviral.2006.04.014 16730806 PMC7114332

[B62] Ho Y.-J. Lu J.-W. Huang Y.-L. Lai Z.-Z. (2019). Palmatine Inhibits Zika Virus Infection by Disrupting Virus Binding, Entry, and Stability. Biochem. Biophysical Res. Commun. 518, 732–738. 10.1016/j.bbrc.2019.08.120 31472967

[B63] Hoever G. Baltina L. Michaelis M. Kondratenko R. Baltina L. Tolstikov G. P. (2005). Antiviral Activity of Glycyrrhizic Acid Derivatives against SARS−Coronavirus. J. Med. Chem. 48, 1256–1259. 10.1021/jm0493008 15715493

[B64] Horgan R. P. Kenny L. C. (2011). 'Omic' Technologies: Genomics, Transcriptomics, Proteomics and Metabolomics. Obstet. Gynaecol. 13, 189–195. 10.1576/toag.13.3.189.27672

[B65] Hossain M. F. Hasana S. Mamun A. A. Uddin M. S. Wahed M. I. I. Sarker S. (2020). COVID-19 Outbreak: Pathogenesis, Current Therapies, and Potentials for Future Management. Front. Pharmacol. 11, 563478. 10.3389/fphar.2020.563478 33178016 PMC7596415

[B66] Hossain M. U. Bhattacharjee A. Emon M. T. H. Chowdhury Z. M. Mosaib M. G. Mourin M. (2021). Recognition of Plausible Therapeutic Agents to Combat COVID-19: An Omics Data Based Combined Approach. Gene 771, 145368. 10.1016/j.gene.2020.145368 33346100 PMC7833977

[B67] Howie B. Marchini J. Stephens M. (2011). Genotype Imputation with Thousands of Genomes. G3. Genes, Genomes, Genet. 1, 457–470. 10.1534/g3.111.001198 PMC3276165 22384356

[B68] Huang K.-L. Lai Y. K. Lin C. C. Chang J. M. (2006). Inhibition of Hepatitis B Virus Production byBoehmeria Nivearoot Extract in HepG2 2.2.15 Cells. World J Gastroenterol. 12, 5721–5725. 10.3748/wjg.v12.i35.5721 17007029 PMC4088177

[B196] Huang S. Chaudhary K. Garmire L. X. (2017). More is Better: Recent Progress in Multi-Omics Data Integration Methods. Front. Genet. 8, 84. 10.3389/fgene.2017.00084 28670325 PMC5472696

[B69] Huang Y.-F. Bai C. He F. Xie Y. Zhou H. (2020). Review on the Potential Action Mechanisms of Chinese Medicines in Treating Coronavirus Disease 2019 (COVID-19). Pharmacol. Res. 158, 104939. 10.1016/j.phrs.2020.104939 32445956 PMC7239792

[B70] Hung M. Lauren E. Hon E. S. Birmingham W. C. Xu J. Su S. (2020). Social Network Analysis of COVID-19 Sentiments: Application of Artificial Intelligence. J. Med. Internet Res. 22, e22590. 10.2196/22590 32750001 PMC7438102

[B198] Infusino F. Marazzato M. Mancone M. Fedele F. Mastroianni C. M. Severino P. (2020). Diet Supplementation, Probiotics, and Nutraceuticals in SARS-CoV-2 Infection: A Scoping Review. Nutrients 12 (6), 1718. 10.3390/nu12061718 PMC7352781 32521760

[B71] Islam M. T. Sarkar C. El‐Kersh D. M. Jamaddar S. Uddin S. J. Shilpi J. A. (2020). Natural Products and Their Derivatives against Coronavirus: A Review of the Non‐clinical and Pre‐clinical Data. Phytotherapy Res. 34, 2471–2492. 10.1002/ptr.6700 32248575

[B72] Israt J. Ahmet O. (2020). Potentials of Plant-Based Substance to Inhabit and Probable Cure for the COVID-19. Turkish J. Biol. 44, 228–241. 10.3906/biy-2005-114 PMC7314514 32595359

[B73] Iwendi C. Bashir A. K. Peshkar A. Sujatha R. Chatterjee J. M. Pasupuleti S. (2020). COVID-19 Patient Health Prediction Using Boosted Random Forest Algorithm. Front. Public Health 8, 357. 10.3389/fpubh.2020.00357 32719767 PMC7350612

[B74] Jin Z. Du X. Xu Y. Deng Y. Liu M. Zhao Y. (2020). Structure of Mpro from SARS-CoV-2 and Discovery of its Inhibitors. Nature 582 (7811), 289–293. 10.1038/s41586-020-2223-y 32272481

[B75] Jo S. Kim H. Kim S. Shin D. H. Kim M. S. (2019). Characteristics of Flavonoids as Potent MERS-CoV 3C-like Protease Inhibitors. Chem. Biol. Drug Des. 94, 2023. 10.1111/cbdd.13604 31436895 PMC7162010

[B76] Jo S. Kim S. Shin D. H. Kim M.-S. (2020). Inhibition of SARS-CoV 3CL Protease by Flavonoids. J. Enzyme Inhib. Med. Chem. 35, 145–151. 10.1080/14756366.2019.1690480 31724441 PMC6882434

[B77] Kabir M. T. Uddin M. S. Hossain M. F. Abdulhakim J. A. Alam M. A. Ashraf G. M. (2020). nCOVID-19 Pandemic: From Molecular Pathogenesis to Potential Investigational Therapeutics. Front. Cel Dev. Biol. 8, 616. 10.3389/fcell.2020.00616 PMC7365855 32754599

[B78] Kanehisa M. Sato Y. (2020). KEGG Mapper for Inferring Cellular Functions from Protein Sequences. Protein Sci. 29 (1), 28–35. 10.1002/pro.3711 31423653 PMC6933857

[B79] Karimi A. Rahim K. A. Rafieian-Kopaei M. (2015). Classification of External Stakeholders Pressures in Green Supply Chain Management. Proced. Environ. Sci. 30 (1), 27–32. 10.1016/j.proenv.2015.10.005

[B80] Khaerunnisa S. Kurniawan H. Awaluddin R. Suhartati S. Soetjipto S. (2020). Potential Inhibitor of COVID-19 Main Protease (Mpro) from Several Medicinal Plant Compounds by Molecular Docking Study. Preprints 2020, 2020030226. 10.20944/preprints202003.0226.v1

[B81] Khan S. A. Zia K. Ashraf S. Uddin R. Ul-Haq Z. (2020). Identification of Chymotrypsin-like Protease Inhibitors of SARS-CoV-2 via Integrated Computational Approach. J. Biomol. Struct. Dyn. 39, 2607–2616. 10.1080/07391102.2020.1751298 32238094

[B82] Kilk K. (2020). Metabolomics Horizon Scanning for 2020: Three Actionable Challenges. Omics: a J. Integr. Biol. 24 (1), 51–52. 10.1089/omi.2019.0194 31834853

[B199] Kim S. Kim D. Cho S. W. Kim J. Kim J. S. (2014). Highly Efficient RNA-Guided Genome Editing in Human Cells Via Delivery of Purified Cas9 Ribonucleoproteins. Genome Res. 24 (6), 1012–1019. 10.1101/gr.171322.113 24696461 PMC4032847

[B83] Kim D. Lee J.-Y. Yang J.-S. Kim J. W. Kim V. N. Chang H. (2020). The Architecture of SARS-CoV-2 Transcriptome. Cell 181 (4), 914–921.e10. 10.1016/j.cell.2020.04.011 32330414 PMC7179501

[B84] Kim D. Min J. Jang M. Lee J. Shin Y. Park C. (2019). Natural Bis-Benzylisoquinoline Alkaloids-Tetrandrine, Fangchinoline, and Cepharanthine, Inhibit Human Coronavirus OC43 Infection of MRC-5 Human Lung Cells. Biomolecules 9, 696. 10.3390/biom9110696 PMC6921063 31690059

[B85] Kiran G. Karthik L. Shree Devi M. S. Sathiyarajeswaran P. Kanakavalli K. Kumar K. M. (2020). In Silico computational Screening of Kabasura Kudineer - Official Siddha Formulation and JACOM against SARS-CoV-2 Spike Protein. J. Ayurveda Integr. Med. 10.1016/j.jaim.2020.05.009 PMC7247487 32527713

[B86] Klenke C. N. (2020). Coronavirus and Environmental Engineering Science. Environ. Eng. Sci. 37, 233–234. 10.1089/ees.2020.0096

[B87] Kodaira H. Spector S. (1988). Transformation of Thebaine to Oripavine, Codeine, and Morphine by Rat Liver, Kidney, and Brain Microsomes. Proc. Natl. Acad. Sci. 85, 1267–1271. 10.1073/pnas.85.4.1267 3422490 PMC279748

[B88] Kotwal G. J. Kaczmarek J. N. Leivers S. Ghebremariam Y. T. Kulkarni A. P. Bauer G. (2005). Anti-HIV, Anti-poxvirus, and Anti-SARS Activity of a Nontoxic, Acidic Plant Extract from the Trifollium Species Secomet-V/anti-Vac Suggests that it Contains a Novel Broad-Spectrum Antiviral. Ann. N.Y Acad. Sci. 1056, 293–302. 10.1196/annals.1352.014 16387696 PMC7167892

[B89] Krishnasamy R. T A. Baba M. Bharath M. V. Phuntsho J. Arunachalam D. (2020). In Silico Analysis of Active Compounds from Siddha Herbal Infusion of Ammaiyar Koondhal Kudineer (Akk) against SARS-CoV-2 Spike Protein and Its ACE2 Receptor Complex. SSRN J. 10.2139/ssrn.3578294

[B90] Kumar R. Sharma A. Haris Siddiqui M. Kumar Tiwari R. (2016). Prediction of Metabolism of Drugs Using Artificial Intelligence: How Far Have We Reached?. Curr. Drug. Metab. 17, 129–141. 10.2174/1389200216666151103121352 26526829

[B91] Kumar R. Sharma A. Iqbal M. S. Srivastava J. K. (2020b). Therapeutic Promises of Chlorogenic Acid with Special Emphasis on its Anti-obesity Property. Curr. Mol. Pharmacol. 13 (1), 7–16. 10.2174/1874467212666190716145210 31333144

[B92] Kumar R. Sharma A. Siddiqui M. H. Tiwari R. K. (2017). Prediction of Human Intestinal Absorption of Compounds Using Artificial Intelligence Techniques. Curr. Drug. Discov. Technol. 14, 244–254. 10.2174/1570163814666170404160911 28382857

[B93] Kumar R. Sharma A. Siddiqui M. H. Tiwari R. K. (2018). Promises of Machine Learning Approaches in Prediction of Absorption of Compounds. Mini. Rev. Med. Chem. 18, 196–207. 10.2174/1389557517666170315150116 28302041

[B94] Kumar R. Sharma A. Srivastava J. K. Siddiqui M. H. Uddin M. S. Aleya L. (2021). Hydroxychloroquine in COVID-19: Therapeutic Promises, Current Status, and Environmental Implications. Environ. Sci. Pollut. Res. 1–14. 10.1007/s11356-020-12200-1 PMC7808930 33447984

[B95] Kumar R. Sharma A. Varadwaj P. Ahmad A. Ashraf G. M. (2012). Classification of Oral Bioavailability of Drugs by Machine Learning Approaches: a Comparative Study. Jcis 2, 1–18. 10.6062/jcis.2011.02.03.0045 22347684

[B96] Kumar R. Srivastava J. K. Singh R. Siddiqui M. H. Mansouri R. A. Abdulhakim J. A. (2020a). Available Compounds with Therapeutic Potential against COVID-19: Antimicrobial Therapies, Supportive Care, and Probable Vaccines. Front. Pharmacol. 11, 1592. 10.3389/fphar.2020.582025 PMC7573470 33123014

[B97] Lalani S. Poh C. L. (2020). Flavonoids as Antiviral Agents for *Enterovirus A71* (*EV-A71*). Viruses 12 (2), 184. 10.3390/v12020184 PMC7077323 32041232

[B98] Lau K.-M. Lee K.-M. Koon C.-M. Cheung C. S.-F. Lau C.-P. Ho H.-M. (2008). Immunomodulatory and Anti-SARS Activities of Houttuynia Cordata. J. Ethnopharmacology 118, 79–85. 10.1016/j.jep.2008.03.018 PMC7126383 18479853

[B99] Letko M. Marzi A. Munster V. (2020). Functional Assessment of Cell Entry and Receptor Usage for SARS-CoV-2 and Other Lineage B Betacoronaviruses. Nat. Microbiol. 5, 562–569. 10.1038/s41564-020-0688-y 32094589 PMC7095430

[B100] Li M. Y. Li L. Zhang Y. Wang X. S. (2020). Expression of the SARS-CoV-2 Cell Receptor Gene ACE2 in a Wide Variety of Human Tissues. Infect. Dis. poverty 9 (1), 45. 10.1186/s40249-020-00662-x 32345362 PMC7186534

[B101] Li T. Peng T. (2013). Traditional Chinese Herbal Medicine as a Source of Molecules with Antiviral Activity. Antiviral Res. 97, 1–9. 10.1016/j.antiviral.2012.10.006 23153834 PMC7114103

[B102] Lin B. Liu J. Liu Y. Qin X. (2020). Progress in Understanding COVID-19: Insights from the Omics Approach. Crit. Rev. Clin. Lab. Sci. 1–18. Advance online publication 10.1080/10408363.2020.1851167 33375876

[B103] Lin K.-T. Wong A. Y.-L. Kaewpreedee P. Sia S. F. Chen D. Hui K. P. Y. (2020). Remdesivir, Lopinavir, Emetine, and Homoharringtonine Inhibit SARS-CoV-2 Replication in vitro. Antiviral Res. 178, 104786. 10.1016/j.antiviral.2020.104786 32251767 PMC7127386

[B104] Loeffelholz M. J. Tang Y.-W. (2020). Laboratory Diagnosis of Emerging Human Coronavirus Infections - the State of the Art. Emerging Microbes *&* Infections 9, 747–756. 10.1080/22221751.2020.1745095 32196430 PMC7172701

[B105] Lu R. Zhao X. Li J. Niu P. Yang B. Wu H. (2020). Genomic Characterisation and Epidemiology of 2019 Novel Coronavirus: Implications for Virus Origins and Receptor Binding. The Lancet 395, 565–574. 10.1016/S0140-6736(20)30251-8 PMC7159086 32007145

[B200] Lubbe A. Verpoorte R. Choi Y. H. (2012). Effects of Fungicides on Galanthamine and Metabolite Profiles in Narcissus Bulbs. Plant Physiol. Biochem. 94, 371–388. 10.1007/s00204-020-02656-y 22804878

[B106] Luo W. Su X. Gong S. Qin Y. Liu W. Li J. (2009). Anti-SARS Coronavirus 3C-like Protease Effects of Rheum Palmatum L. Extracts. Biosci. Trends 3, 124–126. 20103835

[B107] Lurie N. Saville M. Hatchett R. Halton J. (2020). Developing Covid-19 Vaccines at Pandemic Speed. N. Engl. J. Med. 382, 1969–1973. 10.1056/NEJMp2005630 32227757

[B108] Ma R. Yu Z. Cai Q. Li H. Dong Y. Oksman-Caldentey K.-M. (2020). Agrobacterium-mediated Genetic Transformation of the Medicinal Plant Veratrum Dahuricum. Plants 9, 191. 10.3390/plants9020191 PMC7076492 32033134

[B109] Marchini J. Howie B. (2010). Genotype Imputation for Genome-wide Association Studies. Nat. Rev. Genet. 11, 499–511. 10.1038/nrg2796 20517342

[B110] Maurya V. K. Kumar S. Bhatt M. L. B. Saxena S. K. (2020). Therapeutic Development and Drugs for the Treatment of COVID-19. Nat. Public Health Emerg. Collec 2019, 109–126. 10.1007/978-981-15-4814-7_10

[B111] Mazzon M. Marsh M. (2019). Targeting Viral Entry as a Strategy for Broad-Spectrum Antivirals, F1000Res 8, 1628. Faculty Rev-1628 10.12688/f1000research.19694.1 PMC6743247 31559009

[B112] McGuire A. L. Gabriel S. Tishkoff S. A. Wonkam A. Chakravarti A. Furlong E. E. M. (2020). The Road Ahead in Genetics and Genomics. Nat. Rev. Genet. 21 (10), 581–596. 10.1038/s41576-020-0272-6 32839576 PMC7444682

[B113] McKibbin W. J. Fernando R. (2020). The Global Macroeconomic Impacts of COVID-19: Seven Scenarios. SSRN J. 10.2139/ssrn.3547729

[B114] Michaelis M. Doerr H. W. Cinatl J. Jr (2011). Investigation of the Influence of EPs 7630, a Herbal Drug Preparation from *Pelargonium* Sidoides, on Replication of a Broad Panel of Respiratory Viruses. Phytomedicine 18, 384–386. 10.1016/j.phymed.2010.09.008 21036571 PMC7127141

[B115] Miner A. S. Laranjo L. Kocaballi A. B. (2020). Chatbots in the Fight against the COVID-19 Pandemic. Npj Digit. Med. 3, 65. 10.1038/s41746-020-0280-0 32377576 PMC7198587

[B116] Moon K.-B. Park J.-S. Park Y.-I. Song I.-J. Lee H.-J. Cho H.-S. (2019). Development of Systems for the Production of Plant-Derived Biopharmaceuticals. Plants 9, 30. 10.3390/plants9010030 PMC7020158 31878277

[B117] Murray A. Ravichandran K. Singh S. V. Sankar M. Thomas P. Dhama K. (2020). In Silico Molecular Docking Analysis Targeting SARS-CoV-2 Spike Protein and Selected Herbal Constituents. J. Pure Appl. Microbiol. 14, 989–998. 10.22207/JPAM.14.SPL1.37

[B118] Muthuramalingam P. Jeyasri R. Valliammai A. Selvaraj A. Karthika C. Gowrishankar S. (2020). Global Multi-Omics and Systems Pharmacological Strategy Unravel the Multi-Targeted Therapeutic Potential of Natural Bioactive Molecules against COVID-19: An In Silico Approach. Genomics 112, 4486–4504. 10.1016/j.ygeno.2020.08.003 32771622 PMC7410791

[B119] Muller C. Schulte F. W. Lange-Grünweller K. Obermann W. Madhugiri R. Pleschka S. (2018). Broad-spectrum Antiviral Activity of the eIF4A Inhibitor Silve Strol against Corona- and Picornaviruses. Antivir. Res 150, 123–129. 10.1016/j.antiviral.2017.12.010 29258862 PMC7113723

[B120] Naj A. C. (2019). Genotype Imputation in Genome‐Wide Association Studies. Curr. Protoc. Hum. Genet. 102 (1), e84. 10.1002/cphg.84 31216114

[B201] Oliveira R. V. Oliveira M. C. Pelli A. (2017). Disease Infection by Enterobacteriaceae Family in Fishes: A Review. J. Microbiol. Exp. 4 (5), 00128. 10.15406/jmen.2017.04.00128

[B121] Ong E. Wong M. U. Huffman A. He Y. (2020). COVID-19 Coronavirus Vaccine Design Using Reverse Vaccinology and Machine Learning. Front. Immunol. 11, 1581. 10.3389/fimmu.2020.01581 32719684 PMC7350702

[B122] Overmyer K. A. Shishkova E. Miller I. J. Balnis J. Bernstein M. N. Peters-Clarke T. M. (2021). Large-Scale Multi-Omic Analysis of COVID-19 Severity. Cel Syst. 12 (1), 23–40. e7. 10.1016/j.cels.2020.10.003 PMC7543711 33096026

[B123] Pandit M. Latha N. (2020). In Silico studies Reveal Potential Antiviral Activity of Phytochemicals from Medicinal Plants for the Treatment of COVID-19 Infection. Res. Sq. [Epub ahead of print]. 10.21203/rs.3.rs-22687/v1

[B124] Park J.-Y. Yuk H. J. Ryu H. W. Lim S. H. Kim K. S. Park K. H. (2017). Evaluation of Polyphenols from *Broussonetia Papyrifera* as Coronavirus Protease Inhibitors. J. Enzyme Inhib. Med. Chem. 32, 504–512. 10.1080/14756366.2016.1265519 28112000 PMC6010046

[B125] Peng D. Liu X. (2017). Research Advances in Biomarker for Sepsis. Advanced Trauma and Surgery. Editors Fu X. Liu L. (Singapore:Elsevier), 10.1007/978-981-10-2425-2_15

[B126] Peška V. Sitová Z. Fajkus P. Fajkus J. (2017). BAL31-NGS Approach for Identification of Telomeres De Novo in Large Genomes. Methods (San Diego, Calif.) 114, 16–27. 10.1016/j.ymeth.2016.08.017 27595912

[B127] Prasad A. Muthamilarasan M. Prasad M. (2020). Synergistic Antiviral Effects against SARS-CoV-2 by Plant-Based Molecules. Plant Cel Rep 39 (9), 1109–1114. 10.1007/s00299-020-02560-w PMC7303273 32561979

[B128] Prasad A. Prasad M. (2020). SARS-CoV-2: the Emergence of a Viral Pathogen Causing Havoc on Human Existence. J. Genet. 99, 37. 10.1007/s12041-020-01205-x 32482926 PMC7189006

[B129] Qamar U. l. Alqahtani M. T. Alamri S. M. Chen M. A. (2020). Structural Basis of SARS-CoV-2 3CLpro and Anti-COVID-19 Drug Discovery from Medicinal Plants. J. Pharm. Anal. 10, 313–319. 10.1016/j.jpha.2020.03.009 32296570 PMC7156227

[B130] Randhawa G. S. Soltysiak M. P. M. El Roz H. de Souza C. P. E. Hill K. A. Kari L. (2020). Machine Learning Using Intrinsic Genomic Signatures for Rapid Classification of Novel Pathogens: COVID-19 Case Study. PLoS One 15, e0232391. 10.1371/journal.pone.0232391 32330208 PMC7182198

[B131] Rashid M. T. Wang D. (2020). CovidSens: a Vision on Reliable Social Sensing for COVID-19. Artif. Intell. Rev. 54, 1–25. 10.1007/s10462-020-09852-3 PMC7291936 32836651

[B132] Rattanapisit K. Shanmugaraj B. Manopwisedjaroen S. Purwono P. B. Siriwattananon K. Khorattanakulchai N. (2020). Rapid Production of SARS-CoV-2 Receptor Binding Domain (RBD) and Spike Specific Monoclonal Antibody CR3022 in Nicotiana Benthamiana. Sci. Rep. 10 (1), 17698. 10.1038/s41598-020-74904-1 33077899 PMC7573609

[B133] Redka D. y. S. MacKinnon S. S. Landon M. Windemuth A. Kurji N. Shahani V. (2020). PolypharmDB, a Deep Learning-Based Resource, Quickly Identifies Repurposed Drug Candidates for COVID-19. ChemRxiv. 10.26434/chemrxiv.12071271.v1

[B134] Ren J.-l. Zhang A.-H. Wang X.-J. (2020). Traditional Chinese Medicine for COVID-19 Treatment. Pharmacol. Res. 155, 104743. 10.1016/j.phrs.2020.104743 32145402 PMC7128263

[B135] Robson B. (2020). COVID-19 Coronavirus Spike Protein Analysis for Synthetic Vaccines, a Peptidomimetic Antagonist, and Therapeutic Drugs, and Analysis of a Proposed Achilles' Heel Conserved Region to Minimize Probability of Escape Mutations and Drug Resistance. Comput. Biol. Med. 121, 103749. 10.1016/j.compbiomed.2020.103749 32568687 PMC7151553

[B136] Roh C. (2012). A Facile Inhibitor Screening of SARS Coronavirus N Protein Using Nanoparticle-Based RNA Oligonucleotide. Int. J. Nanomedicine 7, 2173–2179. 10.2147/IJN.S31379 22619553 PMC3356205

[B137] Rothan H. A. Byrareddy S. N. (2020). The Epidemiology and Pathogenesis of Coronavirus Disease (COVID-19) Outbreak. J. Autoimmun. 109, 102433. 10.1016/j.jaut.2020.102433 32113704 PMC7127067

[B138] Ryu Y. B. Jeong H. J. Kim J. H. Kim Y. M. Park J.-Y. Kim D. (2010). Biflavonoids from Torreya Nucifera Displaying SARS-CoV 3CLpro Inhibition. Bioorg. Med. Chem. 18, 7940–7947. 10.1016/j.bmc.2010.09.035 20934345 PMC7126309

[B139] Sampangi-Ramaiah M. H. Vishwakarma R. Shaanker R. U. (2020). Molecular Docking Analysis of Selected Natural Products from Plants for Inhibition of SARS-CoV-2 Main Protease. Curr. Sci. 118, 1087–1092.

[B140] Sassi B. A. Harzallah-Skhiri F. Bourgougnon N. Aouni M. (2008). Antiviral Activity of Some Tunisian Medicinal Plants against Herpes Simplex Virus Type 1. Nat. Prod. Res. 22, 53–65. 10.1080/14786410701589790 17999339

[B141] Saxena D. Sharma A. Siddiqui M. H. Kumar R. (2019). Blood Brain Barrier Permeability Prediction Using Machine Learning Techniques: An Update. Curr. Pharm. Biotechnol. 20, 1163–1171. 10.2174/1389201020666190821145346 31433750

[B142] Schnitzler P. Schuhmacher A. Astani A. Reichling J. (2008). *Melissa Officinalis* Oil Affects Infectivity of Enveloped Herpesviruses. Phytomedicine 15, 734–740. 10.1016/j.phymed.2008.04.01810.1016/j.phymed.2008.04.018 18693101

[B143] Sehailia M. Chemat S. (2020). In-silico Studies of Antimalarial-Agent Artemisinin and Derivatives Portray More Potent Binding to Lys353 and Lys31-Binding Hotspots of SARS-CoV-2 Spike Protein Than Hydroxychloroquine: Potential Repurposing of Artenimol for COVID-19. ChemRxiv. 10.26434/chemrxiv.12098652.v1 PMC7441758 32696720

[B144] Semper C. Watanabe N. Savchenko A. (2021). Structural Characterization of Nonstructural Protein 1 from SARS-CoV-2. iScience 24 (1), 101903. 10.1016/j.isci.2020.101903 33319167 PMC7721355

[B145] Semple S. J. Reynolds G. D. O'Leary M. C. Flower R. L. P. (1998). Screening of Australian Medicinal Plants for Antiviral Activity. J. Ethnopharmacology 60, 163–172. 10.1016/s0378-8741(97)00152-9 9582007

[B146] Senior A. W. Evans R. Jumper J. Kirkpatrick J. Sifre L. Green T. (2020). Improved Protein Structure Prediction Using Potentials from Deep Learning. Nature 577, 706–710. 10.1038/s41586-019-1923-7 31942072

[B147] Sepay N. Sepay N. Al Hoque A. Mondal R. Halder U. C. Muddassir M. (2020). In Silico fight against Novel Coronavirus by Finding Chromone Derivatives as Inhibitor of Coronavirus Main Proteases Enzyme. Struct. Chem., 31, 1831, 1840. 10.1007/s11224-020-01537-5 PMC7220622 32412544

[B148] Serkedjieva J. (2003). Influenza Virus Variants with Reduced Susceptibility to Inhibition by a Polyphenol Extract from *Geranium* Sanguineum L. Pharmazie 58, 53–57. 12622254

[B149] Serseg T. Benarous K. Yousfi M. (2020). Hispidin and Lepidine E: Two Natural Compounds and Folic Acid as Potential Inhibitors of 2019-novel Coronavirus Main Protease (2019-nCoVMpro), Molecular Docking and SAR Study. Curr. Comput. Aided Drug Des. 16. 10.2174/1573409916666200422075440 32321407

[B150] Shahriar S. Rana M. S. Hossain M. S. Karim A. Mredula T. N. Nourin N. (2021). COVID-19: Epidemiology, Pathology, Diagnosis, Treatment, and Impact. Curr. Pharm. Des. [Epub ahead of print]. 10.2174/1381612827666210224142446 33655825

[B151] Sharma A. D. kaur i. (2020). Eucalyptol (1,8 Cineole) from *Eucalyptus* Essential Oil a Potential Inhibitor of COVID 19 Corona Virus Infection by Molecular Docking Studies. Preprints. [Epub ahead of print]. 10.20944/preprints202003.0455.v1

[B152] Sharma A. D. Kaur I. (2020). Eucalyptol (1,8 Cineole) from *Eucalyptus* Essential Oil a Potential Inhibitor of COVID 19 Corona Virus Infection by Molecular Docking Studies. Res. Rev. Biotech. Biosci. 7, 59–66. [Epub ahead of print]. 10.20944/preprints202003.0455.v1

[B153] Sharma A. Kumar R. Semwal R. Aier I. Tyagi P. Varadwaj P. (2020). DeepOlf: Deep Neural Network Based Architecture for Predicting Odorants and Their Interacting Olfactory Receptors. IEEE/ACM Trans. Comput. Biol. Bioinf., 1. 10.1109/TCBB.2020.3002154 32750862

[B154] Sharma N. Muthamilarasan M. Prasad A. Prasad M. (2020). Genomics Approaches to Synthesize Plant-Based Biomolecules for Therapeutic Applications to Combat SARS-CoV-2. Genomics 112, 4322–4331. 10.1016/j.ygeno.2020.07.033 32717321 PMC7381398

[B155] Shen L. Niu J. Wang C. Huang B. Wang W. Zhu N. (2019). High-Throughput Screening and Identification of Potent Broad-Spectrum Inhibitors of Coronaviruses. J. Virol. 93, e00023–e00019. 10.1128/JVI.00023-19 30918074 PMC6613765

[B202] Shen F. Fu Z. Y. Wu Y. R. Li L. Zhao Y. D. Xia Y. (2020). Based on Network Pharmacology and High-Throughput Molecular Docking to Study the Potential Molecular Mechanism of Active Compounds That Bind SARS-Cov-2 Specific Target Protein in Jinhua Qingan Granules to Interfere with CovID-19, Modernization of Traditional Chinese Medicine and Materia Materia-World Science and Technology. Available at: http://kns.cnki.net/kcms/detail/11.5699.R.20200421.0949.004.html (Accessed December 20, 2020).

[B156] Shree P. Mishra P. Selvaraj C. Singh S. K. Chaube R. Garg N. (2020). Targeting COVID-19 (SARS-CoV-2) Main Protease through Active Phytochemicals of Ayurvedic Medicinal Plants - Withania somnifera (Ashwagandha), Tinospora cordifolia (Giloy) and Ocimum sanctum (Tulsi) - a Molecular Docking Study. J. Biomol. Struct. Dyn. 1, 1–14. Advance online publication 10.1080/07391102.2020.1810778 PMC7484581 32851919

[B157] Silveira D. Prieto-Garcia J. M. Boylan F. Estrada O. Fonseca-Bazzo Y. M. Jamal C. M. (2020). COVID-19: Is There Evidence for the Use of Herbal Medicines as Adjuvant Symptomatic Therapy? Front. Pharmacol. 11, 581840. 10.3389/fphar.2020.581840 33071794 PMC7542597

[B158] Singh A. Mishra A. (2020). Leucoefdin a Potential Inhibitor against SARS CoV-2 Mpro. J. Biomol. Struct. Dyn. 2020, 1–6. 10.1080/07391102.2020.1777903 PMC7309301 34281489

[B159] Sohrabi C. Alsafi Z. O'Neill N. Khan M. Kerwan A. Al-Jabir A. (2020). World Health Organization Declares Global Emergency: a Review of the 2019 Novel Coronavirus (COVID-19). Int. J. Surg. 76, 71–76. 10.1016/j.ijsu.2020.02.034 32112977 PMC7105032

[B160] Song J. Du L. Li L. Palmer L. C. Forney C. F. Fillmore S. (2015). Targeted Quantitative Proteomic Investigation Employing Multiple Reaction Monitoring on Quantitative Changes in Proteins that Regulate Volatile Biosynthesis of Strawberry Fruit at Different Ripening Stages. J. Proteomics 126, 288–295. 10.1016/j.jprot.2015.06.004 26087350

[B161] Stebbing J. Krishnan V. de Bono S. Ottaviani S. Casalini G. Richardson P. J. (2020). Mechanism of Baricitinib Supports Artificial Intelligence-Predicted Testing in COVID-19 Patients. EMBO Mol. Med. 12 (8), e12697. 10.15252/emmm.202012697 32473600 PMC7300657

[B162] Su Y. Chen D. Yuan D. Lausted C. Choi J. Dai C. L. (2020). Multi-Omics Resolves a Sharp Disease-State Shift between Mild and Moderate COVID-19. Cell 183 (6), 1479–1495 e20. 10.1016/j.cell.2020.10.037 33171100 PMC7598382

[B163] Subramanian I. Verma S. Kumar S. Jere A. Anamika K. (2020). Multi-omics Data Integration, Interpretation, and Its Application. Bioinformatics Biol. insights 14, 1177932219899051. 10.1177/1177932219899051 PMC7003173 32076369

[B164] Suryanarayana L. Banavath D. (2020). A Review on Identification of Antiviral Potential Medicinal Plant Compounds against with COVID-19. Int. J. Res. Eng. Sci. Manage. 3, 675–679.

[B165] Tallei T. E. Tumilaar S. G. Niode N. J. Fatimawali F. Kepel B. J. Idroes R. (2020). Potential of Plant Bioactive Compounds as SARS-CoV-2 Main Protease (Mpro) and Spike (S) Glycoprotein Inhibitors: a Molecular Docking Study. Preprints 2020, 2020040102. 10.20944/preprints202004.0102.v2 PMC7773461 33425427

[B204] Tarazona S. Balzano-Nogueira L. Conesa A. (2018). “Multiomics Data Integration in Time Series Experiments,” in Data Analysis for Omic Sciences: Methods and Applications, Comprehensive Analytical Chemistry. Editors Jaumot J. Bedia C. Tauler R. (Amsterdam, Netherlands:Elsevier) 82, 502–532. 10.1016/bs.coac.2018.06.005

[B166] To K. K. W. Lee K.-C. Wong S. S. Y. Sze K.-H. Ke Y.-H. Lui Y.-M. (2016). Lipid Metabolites as Potential Diagnostic and Prognostic Biomarkers for Acute Community Acquired Pneumonia. Diagn. Microbiol. Infect. Dis. 85, 249–254. 10.1016/j.diagmicrobio.2016.03.012 27105773 PMC7173326

[B167] Tong X. Li A. Zhang Z. Duan J. Chen X. Hua C. (2004). TCM Treatment of Infectious Atypical Pneumonia-Aa Report of 16 Cases. J. Tradit Chin. Med. 24, 266–269. 15688692

[B168] Tsai Y.-C. Lee C.-L. Yen H.-R. Chang Y.-S. Lin Y.-P. Huang S.-H. (2020). Antiviral Action of Tryptanthrin Isolated from *Strobilanthes Cusia* Leaf against Human Coronavirus NL63. Biomolecules 10 (3), 366. 10.3390/biom10030366 PMC7175275 32120929

[B169] Ulasli M. Gurses S. A. Bayraktar R. Yumrutas O. Oztuzcu S. Igci M. (2014). The Effects of *Nigella Sativa* (Ns), *Anthemis Hyalina* (Ah) and *Citrus Sinensis* (Cs) Extracts on the Replication of Coronavirus and the Expression of TRP Genes Family. Mol. Biol. Rep. 41, 1703–1711. 10.1007/s11033-014-3019-7 24413991 PMC3933739

[B170] Umesh U. Kundu D. Selvaraj C. Singh S. K. Dubey V. K. (2020). Identification of New Anti-nCoV Drug Chemical Compounds from Indian Spices Exploiting SARS-CoV-2 Main Protease as Target. J. Biomol. Struct. Dyn. 2020, 1–9. 10.1080/07391102.2020.1763202 PMC7232883 32362243

[B171] Vieira R. F. Bizzo H. R. Deschamps C. (2010). Genetic Resources of Aromatic Plants from Brazil. Isr. J. Plant Sci. 58, 263–271. 10.1560/IJPS.58.2.263

[B172] Wahedi H. M. Ahmad S. Abbasi S. W. (2020). Stilbene-based Natural Compounds as Promising Drug Candidates against COVID-19. J. Biomol. Struct. Dyn. 2020, 1–10. 10.1080/07391102.2020.1762743 32345140

[B173] Wang C. Liu Z. Chen Z. Huang X. Xu M. He T. (2020a). The Establishment of Reference Sequence for SARS‐CoV‐2 and Variation Analysis. J. Med. Virol. 92 (6), 667–674. 10.1002/jmv.25762 32167180 PMC7228400

[B174] Wang D. Yang J. Fan J. Chen W. Nikolic‐Paterson D. J. Li J. (2020b). Omics Technologies for Kidney Disease Research. Anat. Rec. 303 (10), 2729–2742. 10.1002/ar.24413 32592293

[B175] Wang D. Zhang F. Gao G. (2020c). CRISPR-Based Therapeutic Genome Editing: Strategies and *In Vivo* Delivery by AAV Vectors. Cell 181 (1), 136–150. 10.1016/j.cell.2020.03.023 32243786 PMC7236621

[B176] Wang M. Cao R. Zhang L. Yang X. Liu J. Xu M. (2020d). Remdesivir and Chloroquine Effectively Inhibit the Recently Emerged Novel Coronavirus (2019-nCoV) in vitro. Cell Res 30, 269–271. 10.1038/s41422-020-0282-0 32020029 PMC7054408

[B177] Wang X. Dhindsa R. Povysil G. Zoghbi A. Motelow J. Hostyk J. (2020e). Transcriptional Inhibition of Host Viral Entry Proteins as a Therapeutic Strategy for SARS-CoV-2. Preprints 2020, 030360. 10.20944/preprints202003.0360.v1

[B178] Wang Z. Gerstein M. Snyder M. (2009). RNA-Seq: a Revolutionary Tool for Transcriptomics. Nat. Rev. Genet. 10, 57–63. 10.1038/nrg2484 19015660 PMC2949280

[B179] Wang Z. Li L. Yan J. Yao Y. (2020f). Evaluating the Traditional Chinese Medicine (TCM) Officially Recommended in China for COVID-19 Using Ontology-Based Side-Effect Prediction Framework (OSPF) and Deep Learning. Preprints. [Epub ahead of print]. 10.20944/preprints202002.0230.v1 PMC7899032 33631276

[B180] Wink M. (2020). Potential of DNA Intercalating Alkaloids and Other Plant Secondary Metabolites against SARS-CoV-2 Causing COVID-19. Diversity 12, 175. 10.3390/d12050175

[B181] Wishart D. S. (2007). Human Metabolome Database: Completing the 'human Parts List'. Pharmacogenomics 8, 683–686. 10.2217/14622416.8.7.683 18240899

[B203] Woo P. C. Huang Y. Lau S. K. Yuen K. Y. (2010). Coronavirus Genomics and Bioinformatics Analysis. Viruses 2 (8), 1804–1820. 10.3390/v2081803 21994708 PMC3185738

[B182] Xian Y. Zhang J. Bian Z. Zhou H. Zhang Z. Lin Z. (2020). Bioactive Natural Compounds against Human Coronaviruses: a Review and Perspective. Acta Pharmaceutica Sinica. B 10 (7), 1163–1174. 10.1016/j.apsb.2020.06.002 32834947 PMC7278644

[B183] Xu Z. Shi L. Wang Y. Zhang J. Huang L. Zhang C. (2020). Pathological Findings of COVID-19 Associated with Acute Respiratory Distress Syndrome. Lancet Respir. Med. 8, 420–422. 10.1016/S2213-2600(20)30076-X 32085846 PMC7164771

[B184] Yang Y. Islam M. S. Wang J. Li Y. Chen X. (2020). Traditional Chinese Medicine in the Treatment of Patients Infected with 2019-new Coronavirus (SARS-CoV-2): a Review and Perspective. Int. J. Biol. Sci. 16, 1708–1717. 10.7150/ijbs.45538 32226288 PMC7098036

[B185] Yu M.-S. Lee J. Lee J. M. Kim Y. Chin Y.-W. Jee J.-G. (2012). Identification of Myricetin and Scutellarein as Novel Chemical Inhibitors of the SARS Coronavirus Helicase, nsP13. Bioorg. Med. Chem. Lett. 22, 4049–4054. 10.1016/j.bmcl.2012.04.08110.1016/j.bmcl.2012.04.081 22578462 PMC7127438

[B186] Zhang L. Yu J. Zhou Y. Shen M. Sun L. (2020). Becoming a Faithful Defender: Traditional Chinese Medicine against Coronavirus Disease 2019 (COVID-19). Am. J. Chin. Med. 48 (4), 763–777. 10.1142/s0192415x2050038x 32349517

[B187] Zhang Q. Wang Y. Qi C. Shen L. Li J. (2020). Clinical Trial Analysis of 2019‐nCoV Therapy Registered in China. J. Med. Virol. 92, 540–545. 10.1002/jmv.25733 32108352 PMC7228274

[B188] Zhang M.-M. Liu X. M. He L. (2004). Effect of Integrated Traditional Chinese and Western Medicine on SARS: a Review of Clinical Evidence. World. J. Gastroenterol. 10, 3500–3505. 10.3748/wjg.v10.i23.3500 15526373 PMC4576235

[B189] Zhang T. Zhang L. Payne P. R. O. Li F. (2021). Synergistic Drug Combination Prediction by Integrating Multiomics Data in Deep Learning Models. Methods Mol. Biol. (Clifton, N.J.) 2194, 223–238. 10.1007/978-1-0716-0849-4_12 32926369

[B190] Zheng J. Perlman S. (2018). Immune Responses in Influenza A Virus and Human Coronavirus Infections: an Ongoing Battle between the Virus and Host. Curr. Opin. Virol. 28, 43–52. 10.1016/j.coviro.2017.11.002 29172107 PMC5835172

[B191] Zhou G. Li S. Xia J. (2020). Network-Based Approaches for Multi-Omics Integration. Methods Mol. Biol. (Clifton, N.J.) 2104, 469–487. 10.1007/978-1-0716-0239-3_23 31953831

[B192] Zhu N. Zhang D. Wang W. Li X. Yang B. Song J. (2020). A Novel Coronavirus from Patients with Pneumonia in China, 2019. N. Engl. J. Med. 382, 727–733. 10.1056/NEJMoa2001017 31978945 PMC7092803

[B193] Zoppi J. Guillaume J. F. Neunlist M. Chaffron S. (2021). MiBiOmics: an Interactive Web Application for Multi-Omics Data Exploration and Integration. BMC bioinformatics 22 (1), 6. 10.1186/s12859-020-03921-8 33407076 PMC7789220

[B194] Zupanic A. Bernstein H. C. Heiland I. (2020). Systems Biology: Current Status and Challenges. Cell. Mol. Life Sci. 77 (3), 379–380. 10.1007/s00018-019-03410-z 31932855 PMC11104875

